# Archaea express circular isoforms of IS200/IS605-associated ωRNAs

**DOI:** 10.3389/fmicb.2025.1641342

**Published:** 2025-10-14

**Authors:** Beatriz A. Picinato, Vinícius H. Franceschini-Santos, Lívia S. Zaramela, Ricardo Z. N. Vêncio, Tie Koide

**Affiliations:** 1 Departamento de Bioquímica e Imunologia, Faculdade de Medicina de Ribeirão Preto, Universidade de São Paulo, Ribeirão Preto, Brazil; 2 Departamento de Computação e Matemática, Faculdade de Filosofia, Ciências e Letras, Universidade de São Paulo, Ribeirão Preto, Brazil

**Keywords:** circRNA, archaea, RNA-Seq, IS200/IS605, ωRNA, rRNA, tRNA

## Abstract

Circular RNAs (circRNAs) are RNA molecules with 5′ and 3′ ends covalently ligated. Their functions range from acting as genetic regulators to producing proteins, and they are often expressed in a tissue and condition-specific manner. Next-generation sequencing with prior RNA treatment with the RNase R exonuclease (circRNA-Seq) has been used to identify circRNAs in many organisms, especially in model eukaryotes. However, we know little about circRNAs in prokaryotes: they have not been consistently reported in bacteria and, to date, only a few circRNA-Seq studies have been done in archaea. We have developed a prokaryotic-specific computational pipeline, MonArch, that explores RNA-Seq reads for circRNA signatures. We annotated circRNAs in newly generated *Halobacterium salinarum* circRNA-Seq data and reanalyzed over 20 archaeal public RNA-Seq datasets with this tool. *H. salinarum* has 49 high-confidence circRNAs, with some validated by RT-PCR. We detected known circular ribosomal RNA and transfer RNA processing intermediates and novel circRNAs associated with ωRNAs (obligate mobile element–guided activity - OMEGA) and IS200/IS605 transposases. The ωRNAs circular isoforms have a growth-dependent expression pattern, distinct from the total ωRNAs levels. This is one of the few examples of prokaryotic circRNAs with a conditional expression pattern. In all the other public archaea circRNA-Seq data (*Haloferax volcanii, Saccharolobus solfataricus, Sulfolobus acidocaldarius*, and *Pyrococcus abyssi*), we found circRNAs associated with the same classes of transcripts as for *H. salinarum*, including circRNAs in IS200/IS605 transposases in the two *Sulfolobales* species. We broadened our search for circRNAs in representatives of major archaeal groups, and found that circRNAs associated with the rRNA and tRNA are widespread, indicating conserved processing of these transcripts. Circular ωRNAs are also present in other haloarchaeal species. Together, our results show that circRNAs appear to be conserved and abundant among archaea, maybe more than previously appreciated. The circular ωRNAs are present in different distant archaeal species, and are a new piece in the IS200/IS605 system.

## 1 Introduction

Circular RNAs (circRNAs) are RNA molecules with their 5′ and 3′ ends covalently ligated. They are involved in many biological processes in eukaryotes (reviewed in Liu and Chen, 2022) and are long known to be involved in ribosomal (rRNA) and transfer RNA (tRNA) processing in archaea (Kjems and Garrett, 1988; Lykke-Andersen and Garrett, 1994). They were first discovered in viruses and viroids with circular RNA genomes in the 1980s (Sanger et al., 1976; Hsu and Coca-Prados, 1979; Kos et al., 1986). Soon after, “scrambled exons” or “mis-splicing subproducts” were found in human and mouse genes (Nigro et al., 1991; Cocquerelle et al., 1992), but they were dismissed as splicing by-products and non-functional molecules. These exons were, in fact, circRNAs (Cocquerelle et al., 1993), but even with more circRNAs being discovered in the following years, they were not given much attention (Patop et al., 2019).

It was only with the popularization of next-generation sequencing (NGS) and the development of specific bioinformatic tools that circRNAs were described as functional and abundant molecules. They are expressed in a condition and tissue-specific manner (Salzman et al., 2012; Jeck et al., 2013; Memczak et al., 2013), are conserved among different species (Westholm et al., 2014; Rybak-Wolf et al., 2015), and can act as transcriptional (Ashwal-Fluss et al., 2014; Gao et al., 2016; Zhang et al., 2016) and post-transcriptional regulators (Hansen et al., 2013; Memczak et al., 2013; Du et al., 2017). CircRNAs can generate new transcripts (Soma et al., 2007; Birkedal et al., 2020) and proteins (Legnini et al., 2017; Yang et al., 2017); their resistance to exonucleases can make them more stable molecules (Lasda and Parker, 2014). In animals, circRNAs can be associated with aging (reviewed in Cai et al., 2019), autism spectrum disorder (Chen et al., 2020), Alzheimer's disease (reviewed in Zhang et al., 2020), cancer (reviewed in Bach et al., 2019), and viral infections (reviewed in Nahand et al., 2020). The literature on the topic is recent and growing every year.

RNA sequencing specific to find circRNAs (circRNA-Seq) uses RNA treated with RNase R exonuclease to enrich for circRNAs and avoid false positives (Jeck and Sharpless, 2014; Dodbele et al., 2021). This technique has been used extensively in model eukaryotes. However, the explosion in knowledge about circRNAs led by NGS in eukaryotes had no parallel in prokaryotes. Few circRNAs are known in bacteria, with scarce high-throughput initiatives to systematically map them in this domain of life (He et al., 2023). In archaea, only three organisms have circRNA-Seq data published: *Saccharolobus solfataricus* (Danan et al., 2012), *Pyrococcus abyssi* (Becker et al., 2017), and *Haloferax volcanii* (Schwarz et al., 2020). Given the evolutionary implications of the relationship between archaea and eukaryotes (Eme et al., 2018; Williams et al., 2020), it would be important to increase our sampling of diverse archaea to systematically search for circRNAs.

*Halobacterium salinarum* is an archaeon with an established transcriptional regulation network (Brooks et al., 2014) and post-transcriptional regulation information (Lorenzetti et al., 2023). Even with many types of RNAs identified in this organism (Zaramela et al., 2014; Gomes-Filho et al., 2015; Ten-Caten et al., 2018; de Almeida et al., 2019), circRNAs are still a missing part of its regulatory information and network. Insertion sequences (IS) are prokaryotic mobile genetic elements that are post-transcriptionally regulated in *H. salinarum* (Lorenzetti et al., 2023). With 80 full and 33 partial ISs (Siguier et al., 2006; Kichenaradja et al., 2010), they are believed to contribute to *H. salinarum* genome plasticity and instability (DasSarma, 1993; Dulmage et al., 2018). *H. salinarum* ISs from the IS200/IS605 family harbor sense overlapping transcripts (sotRNAs) in their *tnpB* transposase genes (Gomes-Filho et al., 2015) that were generalized for several other Halobacteria in the RFAM database (RFAM families RF02656 and RF02657). Later, it was observed that many other bacteria and archaea had similar transcripts, and this system was implicated as ancestral to the CRISPR/Cas defense mechanism (Kapitonov et al., 2015; Shmakov et al., 2017; Altae-Tran et al., 2021).

The IS200/IS605 transposases (IscB and TnpB) are the ancestral proteins of Cas9 and Cas12 (Kapitonov et al., 2015; Shmakov et al., 2017; Altae-Tran et al., 2021) and were shown to act as RNA-guided endonucleases (Altae-Tran et al., 2021; Karvelis et al., 2021). Both TnpB and IscB have small RNAs (ωRNAs - obligate mobile element-guided activity, OMEGA RNAs) associated with their 3′ or 5′ ends, respectively, that interact with the transposase and guide its activity (Altae-Tran et al., 2021; Karvelis et al., 2021). This system has been tested as a new compact gene editing tool in mammals (Li et al., 2024; Xiang et al., 2024). *H. salinarum* sotRNAs have the same relative position to the *tnpB* as the ωRNAs and also have a conserved structure with ωRNA characteristic features. As such, we will refer to *H. salinarum* sotRNAs as ωRNAs from now on.

In this work, we generated a novel circRNA-Seq dataset for *H. salinarum* and found several circRNAs using a custom-made computational pipeline. The bioinformatics approach is suitable for generic RNA-seq data and could retrieve circRNAs in at least 20 different archaea from public databases. By analyzing our novel and public circRNA-Seq datasets, we could retrieve known circRNAs, as well as annotate new circRNAs associated with the IS200/IS605 family and their ncRNAs, the ωRNAs.

## 2 Materials and methods

### 2.1 *Halobacterium salinarum* growth conditions, RNA extraction, and sequencing

*Halobacterium salinarum* NRC-1 cells were grown in complex media (CM) (NaCl 250g/L (Sigma-Aldrich S9888), MgSO_4_.7H_2_O 20 g/L (Sigma-Aldrich M1880), KCl 2 g/L (Sigma P9541), sodium citrate 3 g/L (Sigma-Aldrich C7254), peptone 10g/L (Oxoid LP0037) (Dyall-Smith, 2009) until OD_600_ ~ 0.5. We extracted small RNAs (<200 nt) using the mirVana miRNA Isolation kit (Ambion). RNA was treated with RNase R as described in (Danan et al., 2012). 120U of RNase R (Biosearch Technologies) was added to 20 μg of RNA for 45 min at 37 °C. The samples were cleaned with the RNeasy MinElute Cleanup kit (QIAGEN) using the Supplementary Protocol “Purification of miRNA from animal cells using the RNeasy^®^ Plus Mini Kit and RNeasy MinElute^®^ Cleanup Kit” protocol 1, where after RLT buffer, 1.5 × volumes of 100% ethanol are added to the sample, applied to the column and eluted in 30 μL of DEPC water. The RNase R treatment was repeated twice, using 3U of RNase R for a μg of purified RNA for subsequent treatments.

We prepared the sequencing library using the TruSeq mRNA Stranded RNA Sample Preparation kit (Illumina), and they were sequenced using the MiSeq Reagent v2 50 cycles kit (Illumina) on the Illumina MiSeq (Illumina). All reactions and preparations were made with two biological replicates.

### 2.2 RNA-Seq data used in reanalysis

We reanalyzed all available archaeal RNase R-treated RNA-Seq available for the identification of circRNAs (as of june 2024). We reanalyzed RNA-Seq data from *Haloferax volcanii* (PRJEB40302) (Schwarz et al., 2020), *Pyrococcus abyssi* (personal communication) (Becker et al., 2017), *Saccharolobus solfataricus* (personal communication) (Danan et al., 2012), and *Sulfolobus acidocaldarius* (PRJNA388657) (Orell et al., 2018).

To make circRNA expression profiles relative to the linear counterpart, we searched for *H. salinarum* circRNAs in RNA-Seq data from different growth stages (López García de Lomana et al., 2020).

We searched for circRNAs in regular RNA-Seq data of representatives of major archaeal groups.

The accession numbers for all data used in this study are in Table S1.

### 2.3 Computational pipeline to identify circRNAs

**Figure 1:**
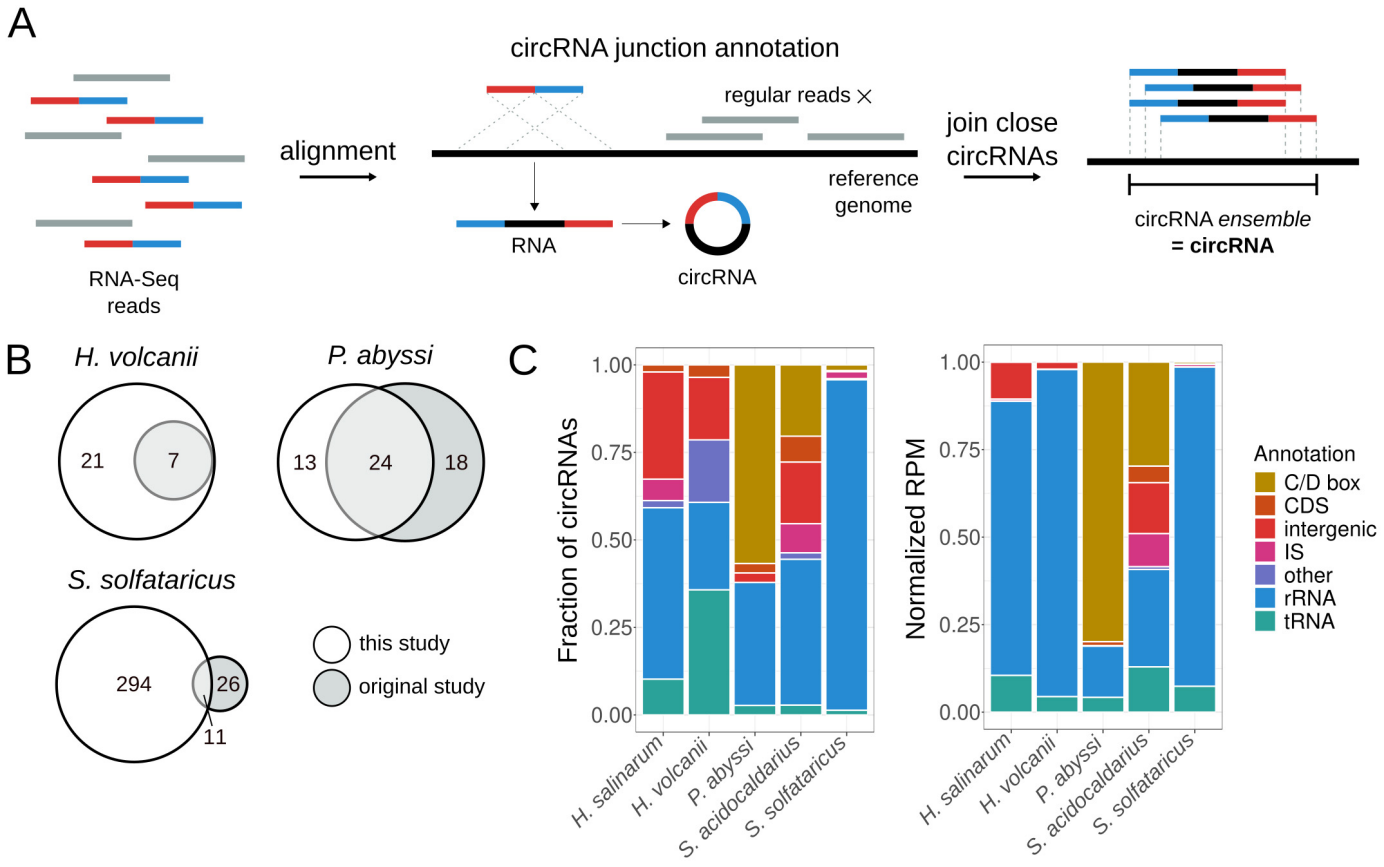
MonArch discovers circRNAs in archaeal circRNA-Seq data. **(A)** Schematic of the MonArch pipeline. First, it aligns the input RNA-Seq reads in the provided reference genome using BLASTn and searches for reads with a chiastic alignment that came from a circularization junction. MonArch then groups close annotated circRNA junctions into one entity, a circRNA *ensemble*, that we call interchangeably as a circRNA. **(B)** Venn diagrams showing how many circRNAs were identified in this study and the original study. **(C)** Categorization of circRNAs annotated by MonArch in all archaeal circRNA-Seq data. Left: fraction of circRNAs in each annotation category. Right: Reads per Million (RPM) counts present in each annotation category, normalized to the circRNA total of each particular study to allow direct comparison. Annotation of circRNAs as in Table S3.

We developed MonArch, a computational pipeline to identify circRNAs in RNA-Seq data. It is based on the premise that reads from the circularization junction align regularly in a chiastic manner in the genome (Figure 1A). The pipeline can be divided into two main parts: (1) identification of individual circularization junctions in the reads and (2) grouping of similar junctions into circRNA *ensembles* (Figure 1A).

MonArch uses the reference genome and the RNA-Seq reads in FASTA format as input; if there are replicates, they are merged into one file. In the first step, the reads are aligned to the reference genome with BLASTn (Altschul et al., 1990), as in other prokaryotic circRNA studies (Danan et al., 2012; Becker et al., 2017). MonArch uses the blastn-short routine with ungapped alignments. Then, a custom Python script searches for a pair of alignments from the same read that could represent a circularization junction to annotate it. The alignments must be uniquely aligned in the genome, do not have mismatches, be no further than 3,500 bases from each other (maximum size we allowed for a circRNA), and together cover at least 90% of the read (Figure S1A). Moreover, the best of the two BLASTn alignments must cover at least half of the read, and the other one should be at least eight bases long (Figure S1A). We allow alignments to have at most a 3nt overlap or gap between them (Figure S1B), as other prokaryotic studies have done before (Danan et al., 2012; Becker et al., 2017). An overlap occurs when a base in the circularization junction can be aligned to the reference genome by either alignment of the pair, while a gap occurs when a base in the circularization junction does not align with the reference genome. The coordinates of the circularization junction are adjusted accordingly.

The pipeline then groups close circularization junctions into a circRNA *ensemble* (Figure 1A). Circularization junctions with their start coordinates distant at most 3nt and end coordinates distant at most 3nt are grouped into one entity. The final coordinates of the circRNA *ensemble* are the minimum among the start coordinates and the maximum among the end coordinates in the forward strand; the reverse is done for the reverse strand. The coordinates of the *ensemble* are not necessarily the same as the coordinates of the junctions that it is made of. We use “circRNA” in the rest of this manuscript to refer to the circRNA *ensemble*.

Some of these parameters can be altered by the user, but for the analyses of this article, we used the default parameters described here for all organisms, with a few exceptions. For all organisms except *H. salinarum*, we used the option “–dont_invert_strand“ because of the nature of the sequencing kits used for each dataset. For *S. solfataricus*, because the sequencing protocol used was not strand-specific, we considered all reads to be on the forward strand after aligning for simplicity before grouping them into circRNA *ensemble*s.

The MonArch pipeline can be found at https://github.com/bpicinato/MonArch.

### 2.4 circRNA identification and annotation in RNA-Seq data

To identify circRNAs in RNA-Seq data, we first trimmed the sequencing adapters (Table S1) from the reads and discarded reads smaller than 20nt using Trimmomatic v0.39 (Bolger et al., 2014). We did not perform this step only in *P. abyssi* data, which was sequenced in an Ion Torrent platform (Thermo Fischer Scientific). Then, if the data was from paired-end sequencing, we grouped the FASTQ R1 and R2 files in one file. Finally, we converted the FASTQ files into FASTA files and used them as input for the MonArch pipeline for circRNA identification. We considered significant circRNAs with 10 or more reads supporting the circularization junction.

Annotation of circRNAs was performed with bedtools intersect (Quinlan and Hall, 2010) using the *ensembles* output BED file containing the circRNAs and a GFF annotation file. Data visualization and integration were done in Integrative Genomics Viewer (Robinson et al., 2011).

To annotate *H. salinarum* NRC-1 circRNAs, we used the gene annotation from (Pfeiffer et al., 2019), the sotRNA/ωRNA annotation from (Gomes-Filho et al., 2015), and the insertion sequence annotation from ISfinder/ISbrowser (Siguier et al., 2006; Kichenaradja et al., 2010).

For *H. volcanii* DS2, we used gene annotation from NCBI (assembly ASM2568v1) and the insertion sequence annotation from HaloLex (Pfeiffer et al., 2008).

For *P. abyssi* circRNAs, we used the gene annotation from NCBI (assembly ASM19593v2), the insertion sequence annotation from ISfinder/ISbrowser (Siguier et al., 2006; Kichenaradja et al., 2010), and the C/D box RNA annotation from (Omer et al., 2000) and (Toffano-Nioche et al., 2013) and the LoweLab (https://lowelab.ucsc.edu/).

For *S. solfataricus* P2, we used the gene annotation from NCBI (assembly ASM1228v1), the insertion sequence annotation from ISfinder/ISbrowser (Siguier et al., 2006; Kichenaradja et al., 2010), and the annotation for non-coding RNAs and C/D box RNAs from (Tang et al., 2005) and (Zago et al., 2005).

For *S. acidocaldarius* DSM639, we used the gene annotation from NCBI (assembly ASM700v1), the insertion sequence annotation from ISfinder/ISbrowser (Siguier et al., 2006; Kichenaradja et al., 2010), the small RNAs annotation from (Orell et al., 2018) and C/D box RNAs from (Omer et al., 2000) and the LoweLab (https://lowelab.ucsc.edu/).

For the other organisms for which RNase R-treated RNA-Seq data do not exist, we used only the gene annotation from NCBI (accession number of assemblies used in Table S1).

### 2.5 Expression profiles of *H. salinarum* circRNAs

We searched for *H. salinarum* circRNAs associated with its ωRNAs and IS200/IS605 in RNA-Seq data of total RNA extracted from different times of a growth curve (López García de Lomana et al., 2020). We considered for analysis circRNAs with at least (i) ten reads supporting the circularization junction in the growth-curve dataset and (ii) one read in the RNase R-treated data. Many ωRNAs had more than one circRNA associated with them; we chose the one with the most read counts across all time points and followed its expression along the growth curve. The coordinates of the circRNAs we analyzed for each ωRNA are in Table S4.

The counts of circRNAs are the counts of how many reads are aligned in their circularization junction. We also counted how many reads aligned in each ωRNA with the featureCounts function from the Rsubread R package (Liao et al., 2019); these are considered the “total reads” (since they are a combination of the reads from linear and circRNA, but not from the circularization junction). The read counts were normalized by the total number of reads sequenced in each time point and corresponding replicate, and multiplied by a million (reads per million, RPM). We calculated the mean and standard error of the three biological replicates for each time point and transcript type (circular or total).

### 2.6 RT-PCR for circRNA experimental validation

Total RNA was extracted from *H. salinarum* NRC-1 grown in the same conditions described in section 2.1 with acid phenol-chloroform (Ambion) and precipitated with ethanol. Small RNAs were extracted with the mirVana miRNA Isolation kit (Ambion). RNase R treatment was performed once with 6U of enzyme (Biosearch Technologies) per μg of RNA. The reaction was cleaned with the RNeasy MinElute Cleanup Kit (QIAGEN). Reverse transcription was done with SuperScript III Reverse Transcriptase (Invitrogen) with random primers and the PCR with GoTaq 2x Master Mix (Promega) using annealing temperatures between 50 °C and 66 °C. We used divergent primers to amplify the circularization junctions (Dodbele et al., 2021). The sequences of the primers used in this study are in Table S2.

### 2.7 *In silico* tools used for RNA structure analysis

The structures of the rRNAs were predicted using R2DT (Sweeney et al., 2021), visualized with RNAcanvas (Johnson and Simon, 2023), and compared with 16S and 23S structures available at Ribovision (Bernier et al., 2014) to determine helix and motif numbering.

We determined the conserved structures of *H. salinarum* ωRNAs using LocARNA with default parameters (Will et al., 2012; Raden et al., 2018). Pseudo-knot structures were predicted with IPknot (v2.2.1) (Sato et al., 2011). The individual structures of the ωRNAs were predicted with Vienna RNAfold (Gruber et al., 2008) and visualized in RNAcanvas (Johnson and Simon, 2023). We removed the base pairing of the predicted guide sequence from the final structures.

### 2.8 Data and code availability

*H. salinarum* raw RNA-Seq data is available at NCBI's Sequence Read Archive (SRA) under the BioProject accession number PRJNA1268524. All the accession numbers for data reanalyzed in this study are in Table S1. The MonArch pipeline is available at https://github.com/bpicinato/MonArch.

## 3 Results

### 3.1 The MonArch pipeline discovers circRNAs in archaea

We developed a computational pipeline, MonArch (https://github.com/bpicinato/MonArch), to identify circRNAs in RNA-Seq data (Figure 1A and Figure S1 for details). MonArch searches for RNA-Seq reads that contain the circularization junction sequence. Reads from these junctions do not align normally in the genome but in a chiastic manner (Figure 1A), a characteristic that allows one to distinguish a circRNA from its linear cognate. MonArch then groups close circRNAs into one entity, as previous works in prokaryotes have done (Figure 1A) (Danan et al., 2012; Becker et al., 2017; He et al., 2023).

We used MonArch to annotate the circRNAs in *H. salinarum* circRNA-Seq data we generated, and reanalyzed all published archaeal circRNA-Seq data to understand circRNA prevalence and distribution among archaea. We reanalyzed data for *H. volcanii* (Schwarz et al., 2020)*, P. abyssi* (Becker et al., 2017)*, S. acidocaldarius* (Orell et al., 2018), and *S. solfataricus* (Danan et al., 2012). For all data analyzed, we considered significant a circRNA with 10 reads or more supporting its circularization junction.

In our reanalysis, MonArch could reasonably recover the results of the original studies, with different success rates for each organism (Figure 1B). It is important to note that different tools or approaches annotate different circRNAs (Hansen et al., 2016; Chen et al., 2021) and that the criteria for the significance of a circRNA vary among all studies. We recovered all the circRNAs originally annotated in *H. volcanii* and annotated 21 new ones associated with tRNAs, the rRNA operon, and the signal recognition particle. For *P. abyssi*, we found the majority (57%) of the circRNAs annotated in the original study and annotated 13 new circRNAs associated with the rRNA operon. Among the circRNAs found only in the original study, 16 (89%) were present in the non-significant circRNAs of our reanalysis. For *S. solfataricus*, we recovered 11 out of the 37 (30%) previously annotated circRNAs and found 294 new circRNAs associated with rRNA, IS, and C/D box small RNAs. 19 (73%) circRNAs found only in the original study were supported by fewer than 10 reads in our analysis. The *S. acidocaldarius* original study did not use the data for circRNA annotation.

For all organisms, MonArch recovered known archaeal circRNAs, such as rRNA and tRNA circular processing intermediates, as well as new circRNAs associated with IS, the rRNA operon, and tRNAs (Figure 1C). The following sections will detail the circRNAs found in *H. salinarum* and other archaea.

### 3.2 MonArch recovers known circRNAs in *Halobacterium salinarum*

We identified 49 high-confidence circRNAs in *H. salinarum* NRC-1 (Table S3). They were associated with three main classes of transcripts: rRNA, tRNA, and IS (Figure 1C). Most of them were associated with the rRNA, followed by intergenic circRNAs, tRNAs, IS, and one circRNA in a hypothetical protein-coding gene (Figure 1C, Table S3). Among them, we could find known circRNAs associated with the 16S rRNA bulge-helix-bulge (BHB) motif, the 5S rRNA, and two circular tRNA introns.

**Figure 2:**
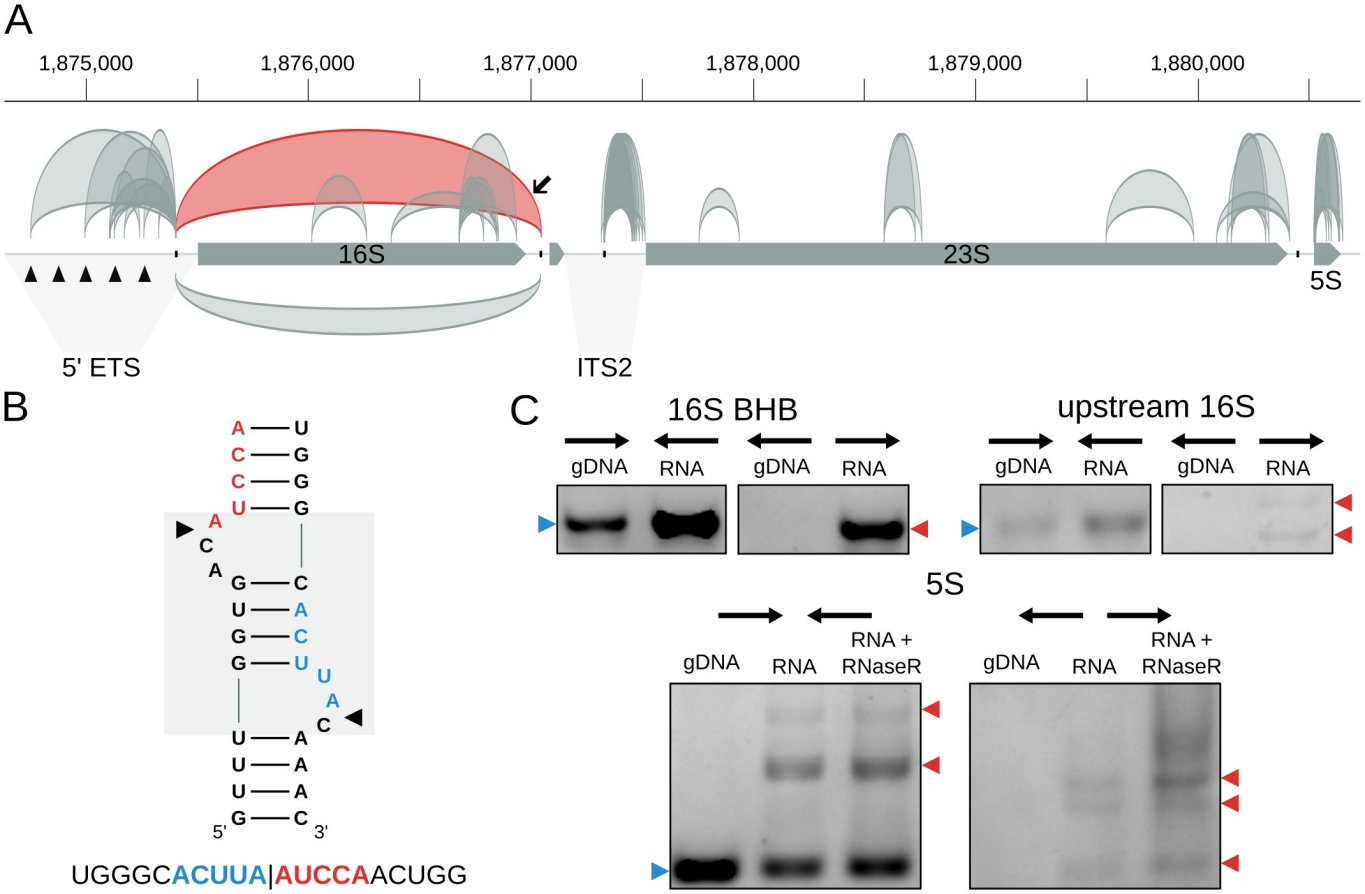
circRNAs in the *Halobacterium salinarum* rRNA operon. **(A)** Scheme of circRNAs in the rRNA operon. Gray arrows represent the genes (16S, tRNA-Ala, 23S, and 5S), while arcs represent the annotated circRNAs with 10 reads or more supporting the circularization junction. On top, circRNAs are in the forward strand; on the bottom, they are in the reverse strand. The circRNA associated with the 16S bulge-helix-bulge (BHB) structural motif is highlighted in red, indicated with a black arrow. Black triangles in the 5′ external transcribed spacer (5′-ETS) region mark the five octanucleotide sequences that are putative transcription start sites. The internal transcribed region (ITS2) is also indicated. The BHB (bulge-helix-bulge) motif positions are indicated with the black ticks in the operon. Coordinates on top of the main chromosome (NC_002607.1), in base pairs. **(B)** 16S BHB structural motif with the most abundant circRNA junction sequence identified below. The gray box in the background highlights the BHB motif, and black arrows mark the canonical processing sites. **(C)** RT-PCR validations of rRNA circRNAs. Convergent arrows represent reactions made with convergent primers (expected to amplify both linear and circular targets), while divergent arrows show reactions with divergent primers (expected to amplify only circular targets). Blue arrows (left) indicate the expected linear product, while red arrows (right) indicate the expected bands for circRNA junction amplification. The multiple arrows in the 16S upstream validation gel indicate expected band sizes for circRNA_0098 and circRNA_0108; multiple arrows in the 5S validation gel indicate different bands made by rolling circle amplification. gDNA = genomic DNA; RNA = reactions made with cDNA amplified from total RNA; RNA + RNase R = reactions made with cDNA made from RNA treated with RNase R. Uncropped images can be found in Figure S4.

The circular processing intermediate associated with the 16S rRNA BHB motif in *H. salinarum* is supported by 2357 reads (circRNA_0143, Table S3, Figure 2A). The pre-23S circular rRNA was supported by 5 reads, probably due to the enrichment for small RNA step performed before RNA sequencing in the *H. salinarum* (Figure S2).

We also identified 7 different circRNAs associated with the 5S rRNA (Table S3, Figures 2A and Figure S3). The one with the most reads (circRNA_0338) encompasses helices II-V, while the second, in the number of reads, encompasses the whole 5S (circRNA_0325) (Figure S3). Interestingly, we found a lot of variability among the start and end coordinates of these circRNAs, especially circRNA_0331 (Figure S3). This might suggest that circularization is a part of the degradation process of the 5S rRNA, as proposed for *S. solfataricus* (Danan et al., 2012).

**Figure 3:**
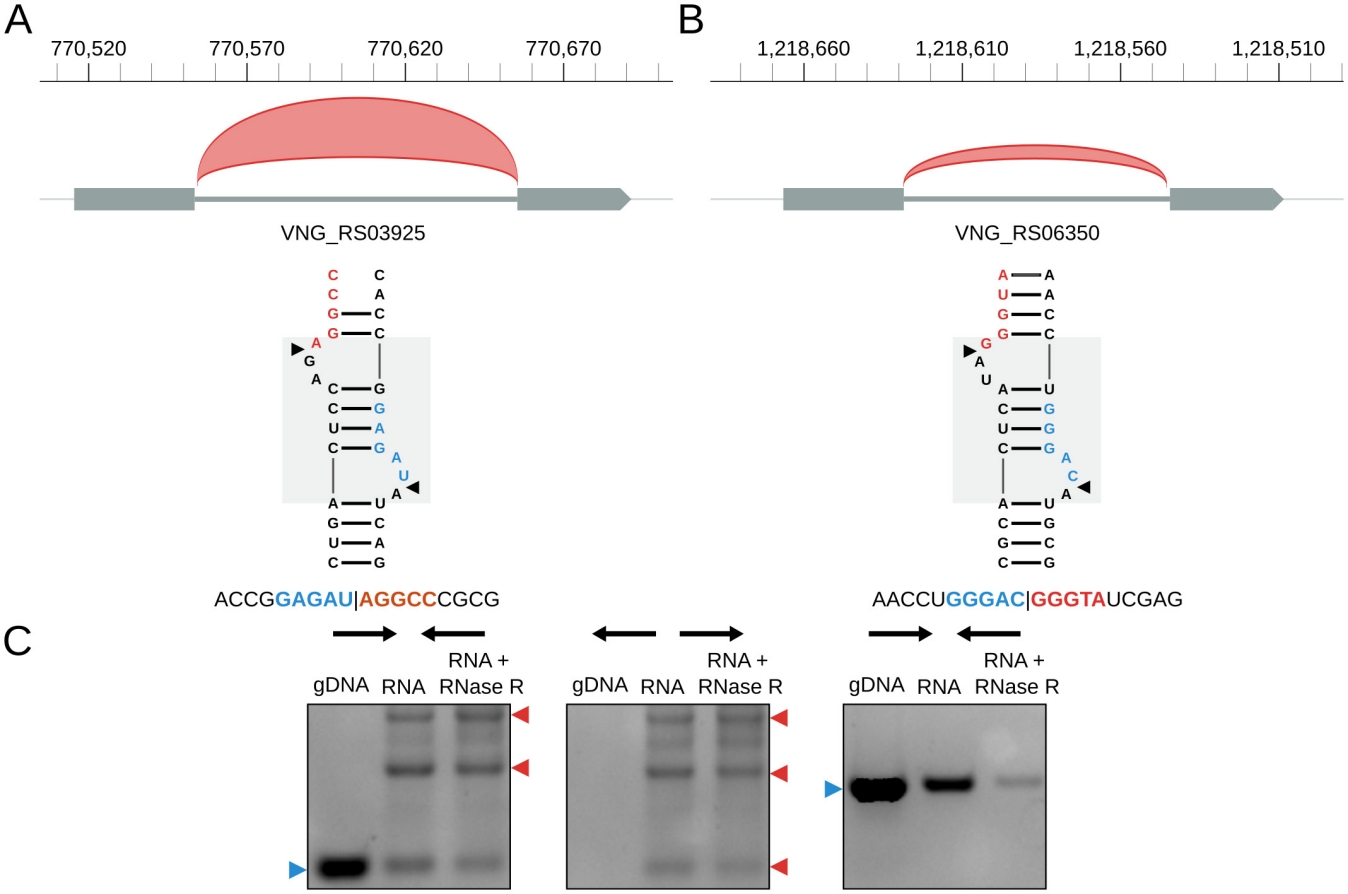
*Halobacterium salinarum* circRNAs in tRNA genes. **(A)** circRNA_0059 (red arc) in tRNA-Trp intron and its associated BHB structural motif. The gray box in the background highlights the BHB motif, and black arrows mark the canonical processing sites. Below is a representative read of this circRNA. Coordinates on top of the main chromosome (NC_002607.1), in base pairs. **(B)** circRNA_0074 (red arc) in tRNA-Met intron and its associated BHB structural motif. The gray box in the background highlights the BHB motif, and black arrows mark the canonical processing sites. Below is a representative read of this circRNA. Coordinates on top of the main chromosome (NC_002607.1), in base pairs. **(C)** RT-PCR validation of the circRNA associated with the tRNA-Trp intron. The left panel is a reaction made with convergent primers expected to amplify both linear and circular templates. The middle panel is the reaction made with divergent primers expected to amplify only circular products. The right panel is made with primers that amplify a linear product that is digested by RNAse R. Blue arrows (left) indicate the expected linear product, while red arrows (right) indicate the expected bands for circRNA junction amplification. The uncut agarose gel is in Figure S7.

*H. salinarum* has two tRNA introns (in tRNA-Trp VNG_RS03925 and tRNA-Met VNG_RS06350); both are circularized at a BHB motif (Figures 3A,B) (circRNA_0059 and circRNA_0074, respectively, in Table S3). tRNA-Trp also has a circRNA with the exact same coordinates as the circular intron but in the opposite strand (circRNA_0060, Table S3). As in *H. volcanii* (Clouet d'Orval et al., 2001) and *P. abyssi* (Omer et al., 2000), *H. salinarum* tRNA-Trp intron is also a C/D box RNA (Weisel et al., 2010).

Using RT-PCR, we validated the circular pre-16S and pre-23S, the circRNAs in the 5S rRNA (Figure 2C, Figure S4), and the tRNA-Trp intron (Figure 3C, Figure S7).

### 3.3 *Halobacterium salinarum* has novel circRNAs

#### 3.3.1 circRNAs associated with the rRNA operon

Besides the canonical rRNA circRNAs, we also found many other smaller circRNAs associated with the rRNA operon. All the circRNAs annotated as “intergenic” (Figure 1C, Table S3) are associated with the rRNA operon upstream of the 16S rRNA or 23S rRNA genes in the 5′ external transcribed spacer 5′-ETS) and internal transcribed spacer 2 (ITS2) (Figure 2A). In the 5′-ETS region, there are five octanucleotide sequences (TGCGAACG) that are putative transcription start sites (Chant and Dennis, 1986). Four of the eight circRNAs in this region start in these sites, and all end at the BHB processing site (Figure 2A). This might suggest that circularization occurs between the 5′ end of the operon transcript and the 3′ left after BHB endonuclease cleavage. These circRNAs were validated using RT-PCR (Figure 2C, Figure S4). In the ITS2 region, all the circRNAs start at the BHB site and end near the start of the 23S gene (Figure 2A, Figure S2).

Inside the 16S and 23S genes, the circRNAs accumulate in their 3′ portions (Figure 2A, Figures S5, S6). In the 16S, most circRNAs are in the 3′M and 3′m domains (Figure S5). In the 23S, the circRNAs are concentrated in the VI domain, predominantly in helices 94-101 (Figure S6).

#### 3.3.2 Circular tRNAs

We found two whole circular tRNAs in addition to the circular tRNA introns: circRNA_0047 in tRNA-Met (VNG_RS02185) and circRNA_0068 in tRNA-Leu (VNG_RS05810) (Figure S8). Their circularization junctions do not have all bases aligned in the reference genome; both have either C, CC, or CCA extra bases between the two halves of the alignment. The sequence “CCA” is formed in the circularization junction in all cases. This exact sequence is added to the 3′ end of the tRNA in its maturation process (Clouet-d'Orval et al., 2018), suggesting circularization occurs after this processing step.

#### 3.3.3 Circular isoforms of IS200/IS605-associated ωRNAs

**Figure 4:**
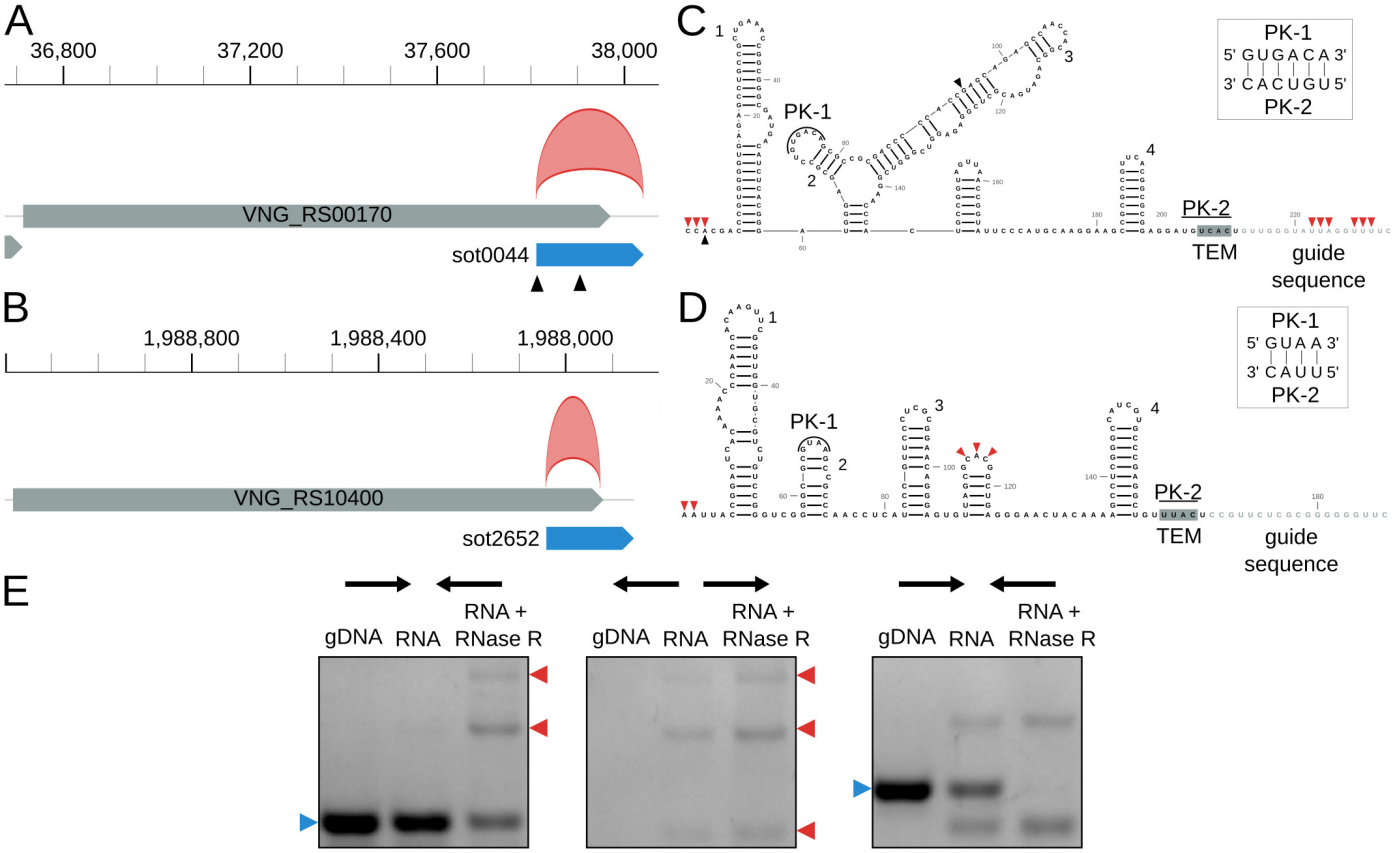
*Halobacterium salinarum* circRNAs in IS200/IS605 transposases and their ωRNAs. **(A)** VNG_RS00170/VNG0044H (gray box) and its ωRNA (blue box) with the annotated circRNA, circRNA_0012 (red arc). Black triangles mark transcript processing sites (TPS) associated with the ωRNA (Ibrahim et al., 2021). Coordinates on top of the main chromosome (NC_002607.1), in base pairs. **(B)** VNG_RS104000/VNG02652H (gray box) and its ωRNA (blue box) with the annotated circRNA, circRNA_0397 (red arc). Coordinates on top of the main chromosome (NC_002607.1), in base pairs. **(C)** RNA structure of sot0044 ωRNA. Black triangles mark the TPS as in panel A. Red triangles mark the start and end of junctions in circRNA_0012. Helices are numbered as in Figure S9. **(D)** RNA structure of sot2652 ωRNA. Red triangles mark the start and end of junctions in circRNA_0012. Helices are numbered as in Figure S9. PK = pseudoknot, TEM = transposon encoded motif. **(E)** RT-PCR validation of circRNA_0397 associated with sot2652. The left panel is a reaction made with convergent primers expected to amplify both linear and circular templates. The middle panel is the reaction made with divergent primers expected to amplify only circular products. The right panel is made with primers that amplify a linear product that is digested by RNAse R. Blue arrows (left) indicate the expected linear product, while red arrows (right) indicate the expected bands for circRNA junction amplification. The uncropped image of the agarose gel is in Figure S11.

Finally, we identified novel circRNAs associated with IS in *H. salinarum*. We found two circRNAs (circRNA_0012 and circRNA_0397, Table S3) associated with the IS200/IS605 family and their ωRNAs (originally named sense overlapping transcripts, sotRNAs, by Gomes-Filho et al., 2015) (Figures 4A, B) and one circRNA in an ISH3/IS4 (Table S3).

*H. salinarum* has 10 ωRNAs with a conserved structure among them (Figure S9). They seem to have characteristic features of previously identified and characterized ωRNAs, such as a pseudoknot between the second hairpin loop and the TEM sequence and a right-end hairpin (Figures 4C,D, Figures S9, S10) (He et al., 2015; Nakagawa et al., 2023; Sasnauskas et al., 2023; Žedaveinyte et al., 2024).

We mapped the circRNAs to the sot0044 and sot2652 ωRNA structures, and we could not find a BHB structural motif associated with them (Figures 4C, D). circRNA_0397 is in the 5′ portion of the RNA, while circRNA_0012 encompasses the whole RNA, including part of the predicted guide sequence. circRNA_0012 coordinates also match a previously annotated RNA processing site (Ibrahim et al., 2021). Both start at the 5′ end of their corresponding ωRNAs.

We validated circRNA_0397 using RT-PCR (Figure 4E, Figure S11).

### 3.4 *H. salinarum* circular ωRNAs have a growth-dependent expression pattern

In eukaryotes, circRNAs can be expressed in a tissue and condition-dependent manner (Salzman et al., 2012; Jeck et al., 2013; Memczak et al., 2013). We searched for circular ωRNAs in other *H. salinarum* RNA-Seq data to understand their expression patterns. We reanalyzed regular RNA-Seq data of *H. salinarum* in different stages of growth (López García de Lomana et al., 2020). Lomana and colleagues sequenced RNA from early (T1, O.D._600_ = 0.2), mid- (T2, O.D._600_ = 0.5), and late (T3, O.D._600_ = 0.8) exponential phases, and from the stationary phase, at 40.8h of growth (T4).

**Figure 5:**
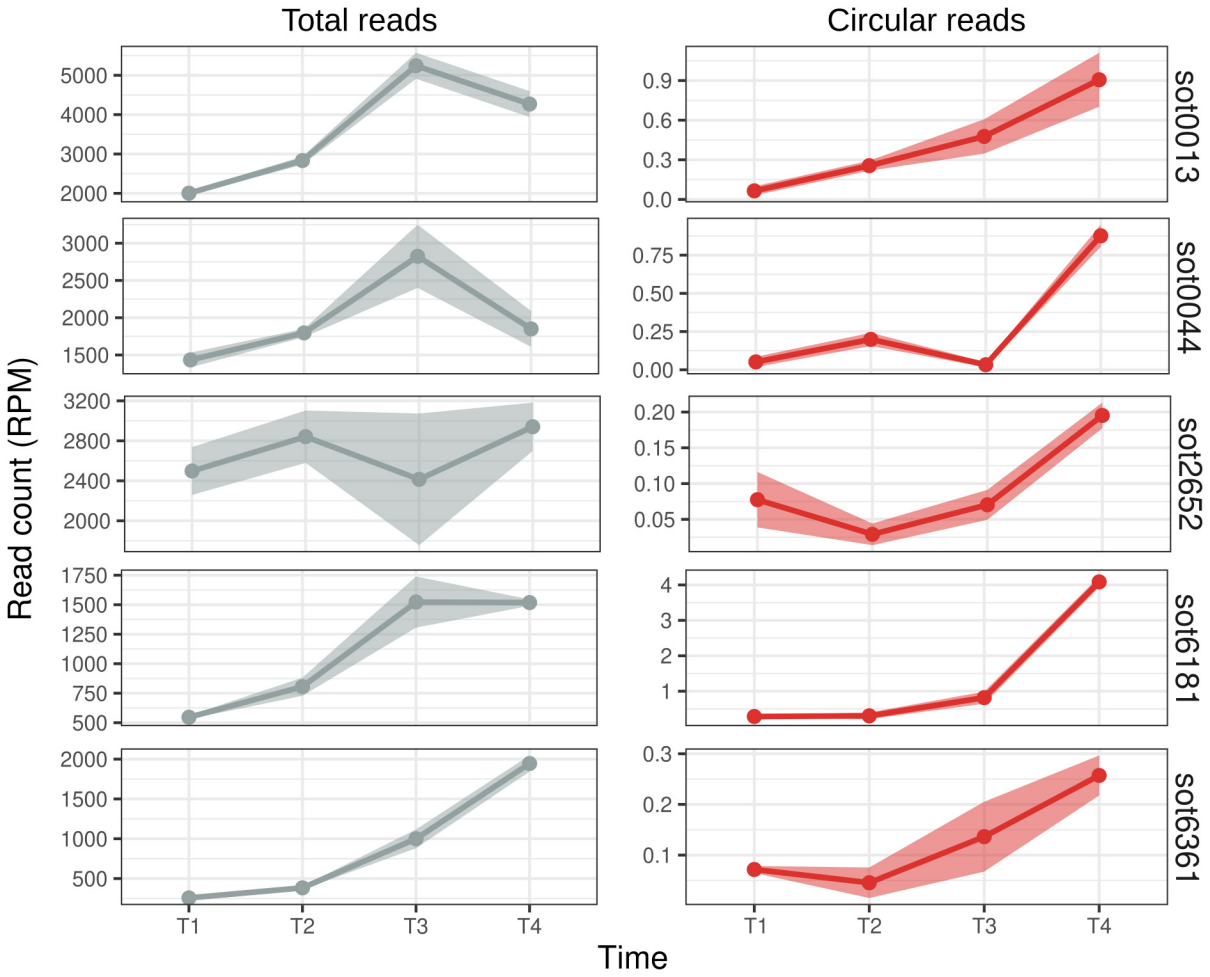
Expression profile of *Halobacterium salinarum* ωRNAs during growth. Read counts (normalized in reads per million - RPM) along the growth curve (T1 = early exponential, T2 = mid-exponential, T3 = late exponential, and T4 = stationary) of *H. salinarum* ωRNAs. Total reads are the reads that aligned normally in the ωRNA locus (left, gray), and circular reads are the reads from the circularization junction (right, red). The mean and standard error of the read counts of 3 biological replicates are shown.

We compared the expression profile of the reads that aligned in the circularization junction, the “circular reads”, and the reads that aligned normally in the ωRNA locus, “total reads”, as they comprise both the reads from the linear isoform and reads that came from the circRNA but not the circularization junction (Figure 5). All the circRNAs analyzed were more expressed in the later stages of growth (Figure 5). They all present different expression patterns and levels among themselves and between the total and circular reads (Figure 5): (i) the total RNA expression is higher in T3, lower in T4, while the circRNA has a peak in T4 (sot0013 and sot0044); (ii) both classes roughly have the same expression pattern (sot6181 and sot6361); and (iii) the total RNA count is fairly constant along the growth curve while the circRNA expression rises from T2 to T4 (sot2652). It is also worth noting that the expression levels of the circular ωRNA do not always correlate with the expression for the whole locus; for example, the highest expressed ωRNA is sot0013 in T3, but the highest expressed circRNA is from sot6181, which has the lowest total read count (Figure 5, Table S4).

It is known that circRNAs can be stable molecules that outlast their linear counterparts (Ashwal-Fluss et al., 2014). This stability could explain why circular ωRNAs are more present in the latter stages of cell growth. However, the counts for each transcript type are in orders of magnitude of difference (Table S4), and in the cell, the circRNAs probably account for a small fraction of the transcripts from ωRNAs. In *Bacillus altitudinis*, circular DucS RNA abundance increases in the latter stages of growth while one of its linear isoforms decreases (He et al., 2023). Together, these two results are the only examples, to our knowledge, of condition-specific expression of prokaryotic circRNAs.

### 3.5 circRNAs are conserved among archaea

In our reanalysis of archaeal circRNA-Seq, we found the same classes of circRNAs we found in *H. salinarum*, in addition to some other already known archaeal circRNAs (Figure 1C, Table S3).

**Figure 6:**
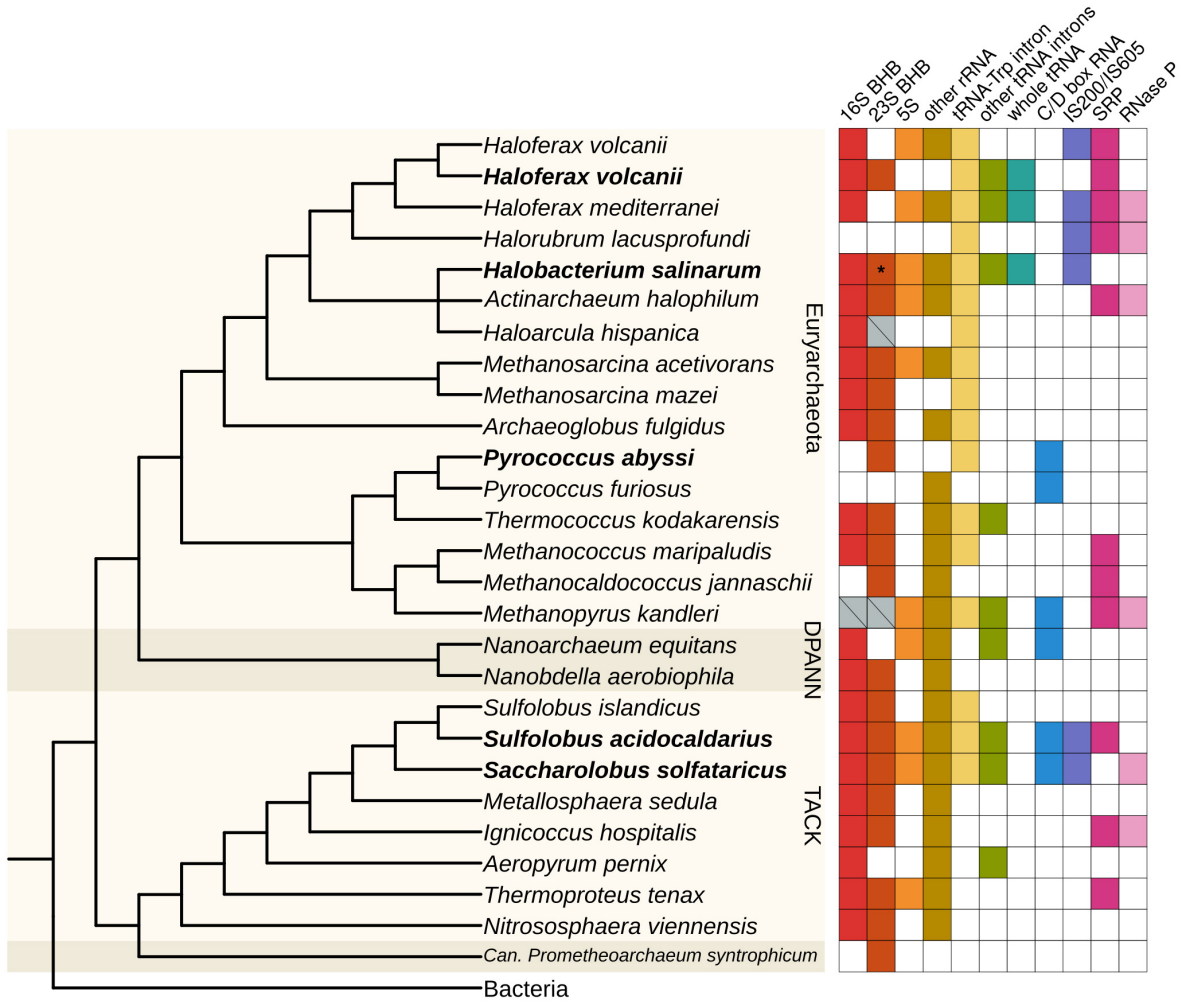
Distribution of circRNAs across archaeal species. The presence of circRNAs in representatives of major archaeal groups was identified by searching for the indicated classes of circRNAs in RNA-Seq data. Organisms with circRNA-Seq data are highlighted in bold. Gray crossed boxes in the “16S BHB” and “23S BHB” categories indicate we could find circRNAs encompassing the whole rRNA gene, being putative circRNAs associated with a BHB motif, but we could not identify or predict the exact BHB structure. *H. volcanii* is duplicated because we used two different datasets to identify circRNAs for this analysis (see Table S1). The asterisk indicates that *H. salinarum* circRNA associated with the 23S BHB does not have 10 reads or more to support it, but it was validated with RT-PCR. The tree was constructed based on the phylogeny from (Liu et al., 2021), with missing organisms added as a polytomy; the Bacteria group were used as an outgroup. BHB, bulge-helix-bulge; SRP, signal recognition particle.

In the circRNA-Seq data, we found circRNAs associated with the rRNA operon and the canonical circular pre-16S and pre-23S in all organisms (*H. volcanii, S. solfataricus*, and *S. acidocaldarius*) except *P. abyssi* (Figure 6, Figure S12). We also found small circRNAs in the rRNA operon in *P. abyssi, S. solfataricus*, and *S. acidocaldarius*. In *P. abyssi*, these circRNAs are concentrated in the 3′ end of the 23S gene, as in *H. salinarum*. In *S. solfataricus*, the circRNAs inside the 16S and 23S genes do not seem to be concentrated in any region in particular. In *S. acidocaldarius*, the small 16S circRNAs are concentrated in its 5′ portion, while in the 23S, they seem to accumulate in domain I. *H. volcanii* has only one small circRNA in the rRNA operon (circRNA_0340, Table S3). We believe that *H. volcanii* is different from the other organisms in this aspect because this was the only dataset that did not have a small RNA enrichment step before sequencing. Both *S. solfataricus* and *S. acidocaldarius* have circRNAs in the 5S rRNA (Figure 6, Figure S13, Table S3).

We identified the circular tRNA-Trp intron for all organisms reanalyzed, besides other circular tRNA introns for *H. volcanii, S. solfataricus*, and *S. acidocaldarius* (Figure 6). In *H. volcanii*, we identified whole circular tRNAs, as we did for *H. salinarum* (Figure 6, Figure S14). These circRNAs also contain some bases in their circularization junctions that do not align in the reference genome, forming the sequence “CCA”.

We also retrieved some previously annotated circRNAs in these archaea. We identified circular C/D box RNAs in the three thermophile species, with this type of circRNA being the most abundant in *P. abyssi* (Figure 1C, Table S3). We also identified circRNAs associated with the signal recognition particle (SRP) in *H. volcanii* and *S. solfataricus*, and with RNase P in *S. solfataricus* (Figure 6, Table S3), as was identified in *S. solfataricus* and *S. acidocaldarius* in the original study (Danan et al., 2012).

We found circRNAs associated with IS200/IS605 in *S. solfataricus* and *S. acidocaldarius* (Figure 6, Table S3). In *S. acidocaldarius*, the circRNAs are associated with the 3′ of the transposase gene, while in *S. solfataricus*, they are in the 5′ end. In both species, the RNA-Seq coverage in the transposase is consistent with the presence of an ωRNA (Figure S14). *S. solfataricus*, besides circRNAs in two IS200/IS605 transposases, has circRNAs associated with three IS not classified yet (ISNCY) (Table S3).

Motivated by the finding of circular ωRNAs in other archaea and that circRNAs appear to be conserved among different species, we expanded our search for circRNAs in representative species of major archaea groups (Figure 6). Using regular RNA-Seq data, we searched for the same types of circRNAs we identified in RNase R-treated data to avoid false positives, and used 10 reads supporting the circularization junction as a cutoff to consider a circRNA significant.

The most consistent classes of circRNAs identified in all datasets are circRNAs in rRNA and tRNA (especially tRNA-Trp intron), indicating circularization is an important or at least a conserved part of processing these transcripts (Figure 6). Indeed, in the circRNA-Seq datasets, these circRNAs were the most abundant in most organisms studied (Figure 1C). We found whole circular tRNAs only in haloarchaea species (*H. salinarum, H. volcanii*, and *Haloferax mediterranei*), indicating this could be haloarchaea-specific processing (Figure 6). We found circular C/D box RNAs (not considering tRNA introns) only in thermophile species (Figure 6), which supports the hypothesis that the circularization of these transcripts is important at high temperatures (Starostina et al., 2004).

We found circRNAs associated with IS200/IS605 in *H. mediterranei, H. volcanii*, and *Halorubrum lacusprofundi* (Figure 6). These three species are haloarchaea belonging to the Haloferacales order. In the RFAM database, the RFAM families RF02656 and RF02657 (still bearing the original sotRNA terminology) from *H. salinarum* ωRNAs were expanded to other Halobacteria, indicating that ωRNAs, and probably their circular isoforms, might be abundant in these groups.

## 4 Discussion

### 4.1 MonArch consistently identifies archaeal circRNAs

MonArch, our computational pipeline for finding circRNAs in RNA-Seq data, showed good results in recovering known archaeal circRNAs in all circRNA-Seq datasets and in our reanalysis compared with published results (Figures 1B, C). MonArch assumes that reads from the circularization junction do not align regularly in the genome but in a chiastic manner. Even though simple, this approach has proven effective in searching for prokaryotic circRNAs.

MonArch does not need major data pre-processing before using it to annotate circRNAs. Other eukaryotic-focused tools need the RNA-Seq data to be aligned by specific tools before the identification of the circRNAs. MonArch can handle raw RNA-Seq reads as well as reads that have been processed in some manner, as long as they are in FASTA format. In this study, we chose to first trim adapters from the RNA-seq reads since it helped MonArch identify circRNAs downstream. We also used unaligned reads from an RNA-Seq read aligner in our tests. This could save some time in processing large datasets, but the filters in MonArch itself can identify circRNAs with confidence (Figure S1A), making it not mandatory. This approach resulted in the same circRNAs reported here but with fewer reads supporting each one (results not shown).

MonArch also does not need any other information on the organism studied besides its reference genome. Published tools often need genome annotation or splicing annotation for the organism. This seems to be useful in the precise annotation of high-confidence circRNAs in eukaryotes (Vromman et al., 2023), but it can be a hindrance to use in prokaryotes that do not have splicing events or well-annotated genomes. However, even tools that support *de novo* circRNA annotation cannot identify prokaryotic circRNAs adequately. We have tested CIRI2 (Gao et al., 2015, 2018) and circRNA_finder (Westholm et al., 2014) with default parameters in the *H. salinarum* circRNA-Seq dataset. CIRI2 only identified one circRNA, while circRNA_finder identified none. Another study also tested CIRI2 in their *S. solfataricus* RNA-Seq data, and it could not retrieve all the circRNAs they validated (Bathke et al., 2020).

However, it is important to stress that MonArch was made to analyze prokaryotic RNA-Seq data. It was never tested or optimized for larger eukaryotic datasets and genomes. Besides, it only searches for the circularization junction signature and will not necessarily benefit from paired-end sequencing information when reads come from different sides of the junction but do not contain it. Finally, given its simplicity and standard tools, it could be a prohibitively slow approach for facility-level workloads.

### 4.2 Biogenesis of circular ωRNAs

In archaea, it is well-known that circRNAs can be generated by processing the RNA at the bulge-helix-bulge (BHB) RNA motif by EndA, an endonuclease, followed by ligation of the ends (Clouet-d'Orval et al., 2018; Qi et al., 2020; Schwarz et al., 2020; Grünberger et al., 2023). The ligation reaction could be performed by RtcB or other RNA ligases since Pab1020, a ligase from the Rnl3 family, has been found to circularize RNAs in *P. abyssi* (Becker et al., 2017). However, we did not find the BHB motif associated with all *H. salinarum* circRNAs, in particular the circular ωRNAs (Figures 4C, D). This suggests these circRNAs may have a different biogenesis pathway independent of the BHB motif and EndA endonuclease, as was proposed before for other archaeal circRNAs (Danan et al., 2012; Becker et al., 2017). This is not surprising, since all domains of life seem to circularize their RNAs by different pathways, and even RNAs of one domain have different means to do so (with the pairing of flanking Alu repeats or binding of RNA-binding proteins in Eukarya (Kristensen et al., 2019), and self-splicing introns (Hausner et al., 2014; Roth et al., 2021) or the independent mechanism of DucS circularization in Bacteria (He et al., 2023), for example).

TnpBs can process their own ωRNAs at the 5′ end, according to a survey of 59 TnpB orthologs with an *in vitro* transcription and translation system (Nety et al., 2023). Ibrahim and colleagues also observed transcriptional processing sites (TPS) at IS200/IS605 of different prokaryotic organisms, further suggesting that processing of the ωRNA is a widespread phenomenon (Ibrahim et al., 2021).

Gomes-Filho and colleagues' Northern-Blot experiments suggest *H. salinarum* ωRNAs/sotRNAs are processed from the primary transcript (Gomes-Filho et al., 2015). Also, its ωRNAs are enriched for TPS, especially at their 5′ end (Ibrahim et al., 2021; Lorenzetti et al., 2023). These suggest the processing of the ωRNA from the *tnpB* RNA in *H. salinarum*. Given that all of the *H. salinarum* circular ωRNAs we studied here (identified in the RNase R-treated and the growth curve datasets) start at the 5′ end of their cognate linear ωRNA, it is reasonable to assume TnpB could process the transcript for circularization. However, it remains unclear if TnpB or other RNA nuclease processes the RNA at the 3′ end. In the case of the circularization of the whole or majority of ωRNA (as circRNA_0012, Figures 4A, C), it could be the case that circularization occurs between the newly processed 5′ end of the ωRNA and the 3′ of the transcript. It is still unknown which RNA ligase could ligate the circRNA 5′ and 3′ ends for these transcripts.

### 4.3 Possible functions of circular ωRNAs

We found novel circRNAs associated with IS200/IS605 and their ωRNAs in *H. salinarum* and other halophilic archaea, as well as in *S. solfataricus* and *S. acidocaldarius*. The interest in IS200/IS605 transposases and their ωRNA has been increasing in the last few years due to their evolutionary relationship to Cas9 and Cas12 (Kapitonov et al., 2015; Shmakov et al., 2017; Altae-Tran et al., 2021) and the promise of a new and more compact gene-editing tool (Li et al., 2024; Xiang et al., 2024). The circRNAs associated with the ωRNAs are a new piece in this puzzle with unexplored functions and biogenesis pathways.

The growth-dependent expression of the circular ωRNAs might suggest some function or regulation in the cell. Bacterial circular DucS regulates the level of its linear counterpart: in later stages of growth, the circular isoform has increased expression, while the level of the linear isoform decays (He et al., 2023). *H. salinarum* sot0013 and sot0044 ωRNAs have an expression pattern that could suggest this kind of regulation (Figure 5), but more orthogonal approaches are necessary to check this hypothesis. *H. salinarum tnpBs* and their ωRNAs have inverse expression profiles (Gomes-Filho et al., 2015), and its TnpB proteins are either detected at very low levels or not detected at all by mass spectrometry (Lorenzetti et al., 2023).

*H. salinarum* mobilome and transposition are post-transcriptionally regulated by different mechanisms (Lorenzetti et al., 2023). We searched for *H. salinarum* circular ωRNAs in different RNA-Seq datasets besides the growth curve shown in Figure 5 (low salinity, different genetic backgrounds), but we either (i) did not find evidence for circular ωRNAs or (ii) could not see an expression profile for the total reads that was different for the circular ones in these datasets (data not shown). This could be due to the lack of RNase R enrichment and consequent inability to consistently detect circRNAs and to the actual absence of circRNA regulation in the conditions investigated.

Transposition and transposases are usually kept at low levels in the cell to avoid deleterious effects on the host (Ellis and Haniford, 2016). This is achieved by a myriad of mechanisms at different information levels (Nagy and Chandler, 2004). Specifically in the IS200/IS605 family, both the transposition by TnpA and the DNA cleavage by TnpB seem to be post-transcriptionally regulated (Ellis et al., 2015; Nety et al., 2023). Post-transcriptional regulation fine-tunes gene expression in a condition-dependent manner allowing rapid adaptation to stress (Martínez and Vadyvaloo, 2014; Papenfort and Melamed, 2023) through RNA processing, stability, and turnover (Shine et al., 2024). It has been proposed that circRNAs could also be part of the RNA degradation process (Danan et al., 2012), and IS200/IS605 circRNAs (one of which we reidentified here—circRNA_7379 in Table S3) interact with exosome proteins in *S. solfataricus* (Bathke et al., 2020).

circRNAs have the exact same sequence as their linear counterpart (except for the circularization junction), and yet they can have different conformations and structures (Chen, 2016), which could lead to different functions. The prediction of the circular or linear ωRNA in Vienna RNAfold (Gruber et al., 2008) did not significantly change the structure shown in Figures 4C, D. Since Cryo-EM experiments have shown that 2D structure predictors could not accurately reproduce the experimentally found structure for the ωRNA (Nakagawa et al., 2023; Sasnauskas et al., 2023), it is reasonable to assume that this does not necessarily mean that the circular and linear ωRNA have the same structure.

In eukaryotes, some circRNAs form short imperfect duplexes absent in their linear counterparts that can bind to proteins and modulate their activity. For example, circRNAs can regulate the innate immune response by binding to the dsRNA-binding PKR (protein kinase R) (Liu et al., 2019) or the DNA-binding protein cGAS (cyclic GMP-AMP synthase) to avoid its self-DNA activity (Xia et al., 2018). If circular and linear ωRNAs have different structures, the circRNA could have a similar role with TnpB, serving as a decoy to avoid the protein activity when unnecessary. The interaction with exosome proteins in *S. solfataricus* may suggest a role in degradation or degradation signaling for these circRNAs; in eukaryotes, circRNA conformation and interaction partners are signals to modulate its turnover (Liu et al., 2024).

Yet, the actual functions and structural conformation of circular ωRNAs remain to be properly elucidated. The first steps in this endeavor could be to determine the interaction partners of both the circular and linear ωRNA, and eventually experimentally determine their structures. Classical genetic and functional approaches are also important, but careful planning and controls are necessary to address the challenge of overlapping transcripts (in this case, the *tnpB* mRNA and the linear and circular forms of the ωRNA). Studies on the circularization mechanisms of the ωRNA would also be of great help for informing the genetic manipulations in these loci.

### 4.4 Conservation of circRNAs among archaea

Using circRNA-Seq data, we found circRNAs associated with the same classes of transcripts in all organisms studied (rRNA, tRNA, IS, C/D box RNA, and other small RNAs) (Figure 1C). This is evidence that circRNAs are conserved among this group of organisms since they are associated with the same transcripts. More than that, in our expanded search for circRNAs in archaea representatives, we could find at least one circRNA in each of them, even without RNase R enrichment (Figure 6). Our search identified the novel circular ωRNAs in different phylogenetically distant species, indicating this could be a widespread or conserved phenomenon.

It is important to point out that our results on the presence of circRNAs do not necessarily result only from phylogenetic relationships. Even though we searched only for circRNAs annotated in circRNA-Seq data in regular RNA-Seq datasets to avoid false positives, the latter were not treated with RNase R and probably did not consider circRNA annotation when they were made. This leads to a great variability in results and what can be detected according to how the data was generated. For example, the sequencing of small RNAs may prevent the identification of circular pre-16S and pre-23S, very established and known circular intermediates in archaea. The opposite may also be true, with total RNA sequencing hindering smaller circRNA detection. The identification of circular ωRNAs in one *H. volcanii* dataset and not the other (total RNA with no rRNA depletion, treated with RNase R vs. RNA-Seq of small RNAs) (Figure 6) exemplifies this limitation. The sequencing depth may also influence whether less abundant circRNAs are detected, especially without RNase R treatment.

Considering this, we do not believe that our results represent a final landscape of circRNA presence in archaea. If a circRNA is absent in one of the organisms in this analysis, it does not necessarily mean it does not exist. Our analysis is a first effort, using already published data, to detect archaeal circRNAs in a more comprehensive and systematic way. We believe that many more circRNAs are to be identified in Archaea (even more examples of circular ωRNAs); we show here that circRNAs are present in all groups investigated, making future circRNA searches promising.

### 4.5 Concluding remarks

Here, we have identified and described in detail *H. salinarum* circRNAs using MonArch, a computational pipeline we developed for circRNA identification in archaeal RNA-Seq data. We could find known circRNAs as rRNA and tRNA processing intermediates, as well as new circRNAs associated with IS200/IS605 and their non-coding RNAs, ωRNAs. We also showed that these circRNAs are expressed in a growth-dependent manner in *H. salinarum*, being one of the few examples of circRNA conditional expression in prokaryotes. These circRNAs could be interesting new pieces in the TnpB/ωRNA system.

With our extensive reanalysis of RNA-Seq data, we showed that the same classes of transcripts are circularized in archaea. circRNAs seem to be prevalent and conserved in this group of organisms, maybe more than previously appreciated. The circular ωRNAs also seem to be conserved, being present in haloarchaea and two Sulfolobales species, two phylogenetically distant archaeal groups.

With this study, we contributed to expanding the yet scarce knowledge of prokaryotic circRNAs. We hope our results incentivize the search and characterization of circRNAs in other archaea to gain a better understanding of these molecules in the third domain of life and in prokaryotes as a whole.

## Data Availability

The datasets presented in this study can be found in online repositories. The names of the repository/repositories and accession number(s) can be found in the article/Supplementary material.
