# Supplementary material for: Archaea express circular isoforms of IS200/IS605-associated ωRNAs
Source: Front Microbiol. 2025 Oct 14;16:1641342. doi: 10.3389/fmicb.2025.1641342 (PMC12558985; doi:10.3389/fmicb.2025.1641342)
Supplement: Supplementary file 5 [file Data_Sheet_1.pdf]

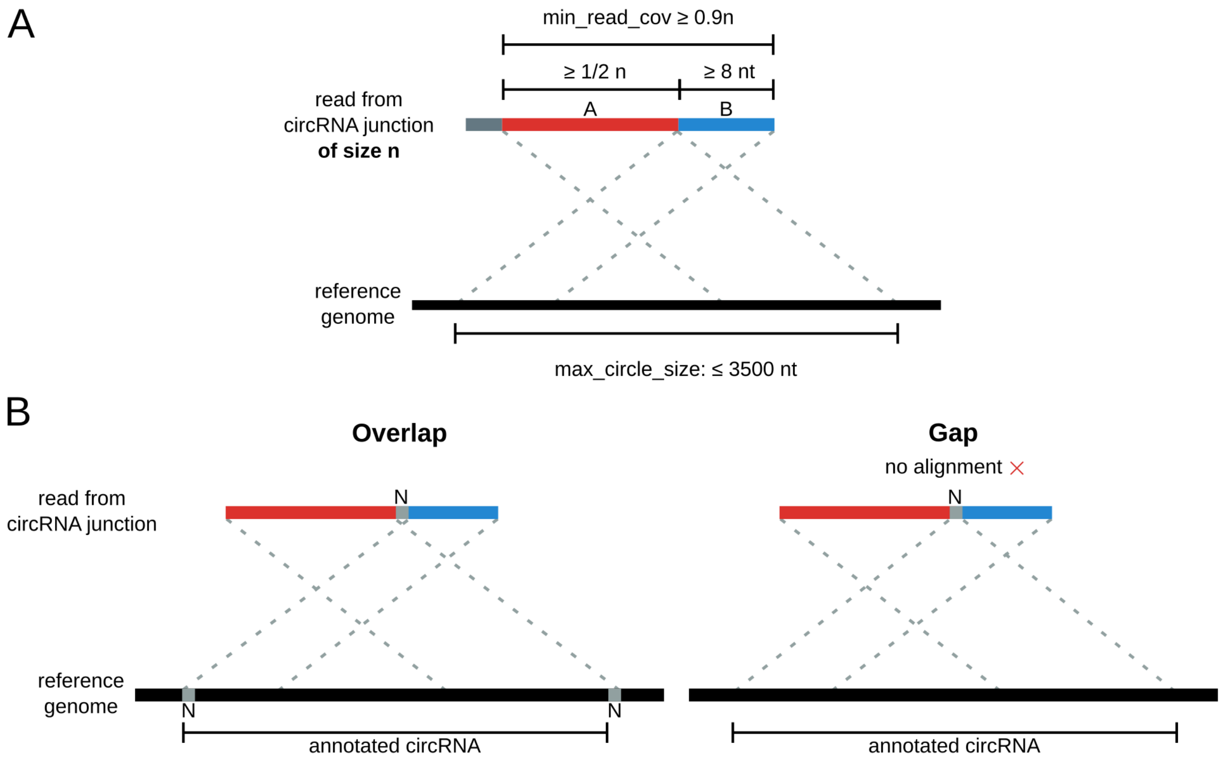

Figure S1: Details on the MonArch pipeline. (A) Schematic of some MonArch parameters to identify circularization junctions in RNA-Seq reads. We considered a valid junction for circRNA annotation if: the best of the BLASTn alignments (i) cover at least half of the read, and the other one ("second hit", ii) is at least eight bases long; the alignments are uniquely aligned in the genome (no multi-mappers), each of the halves alignments (i and ii) do not have mismatches in the reference genome, are no further than 3500 bases from each other (max_circle_size), and together cover at least 90% of the read (min_read_cov). **(B)** Reads that contain a circularization junction are allowed to have at most a 3nt "overlap" or "gap" between the two halves of the alignment. An overlap (left) occurs when a base (N) can be aligned to either side of the transcript. The final circRNA coordinate always considers that the base came from the 5' portion of the circularized transcript. A gap (right) occurs when there is a base (N) between the two portions of the alignment that does not align to the reference genome.

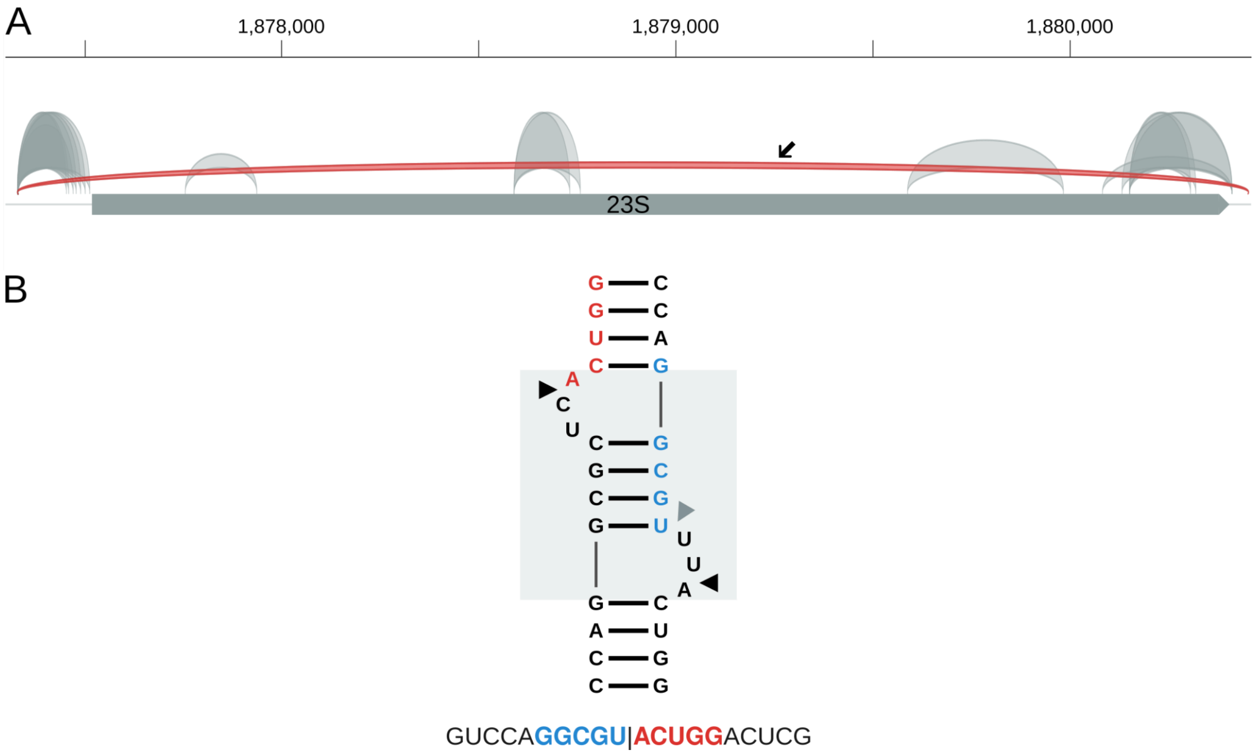

Figure S2: circRNA in the 23S BHB. (A) Scheme of circRNAs in the 23S gene. Arcs represent the annotated. The circRNA associated with the 23S bulge-helix-bulge (BHB) structural motif is highlighted in red, indicated with a black arrow. Coordinates on top of the main chromosome (NC_002607.1), in base pairs. **(B)** 23S BHB structural motif with the identified circRNA junction sequence is shown below. The gray box in the background highlights the BHB motif, black arrows mark the canonical processing sites, and the gray arrow marks where processing should occur based on the circRNA read we identified.

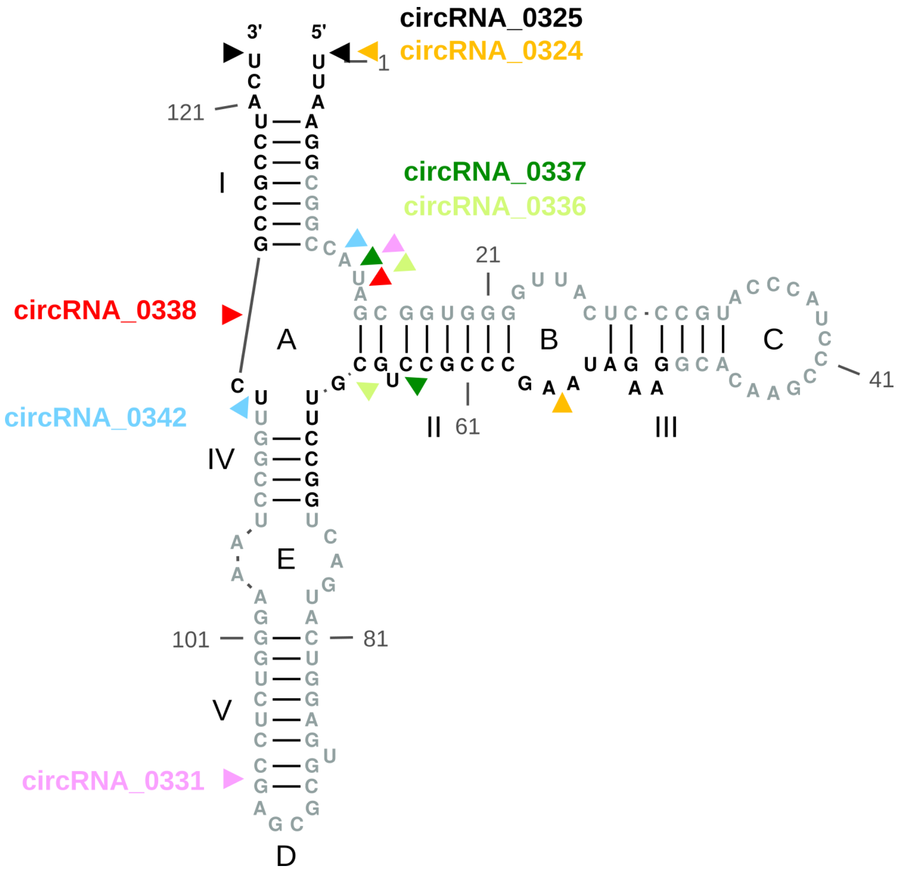

Figure S3: *Halobacterium salinarum* 5S rRNA structure. The start and end of the most common read in each circRNA are marked in the colored arrows. Nucleotides that are the start or end of some read comprising circRNA_0331 are in gray. Roman numerals indicate 5S helix structures, and letters indicate the loops. circRNA_0338 is the most abundant, followed by circRNA_0325.

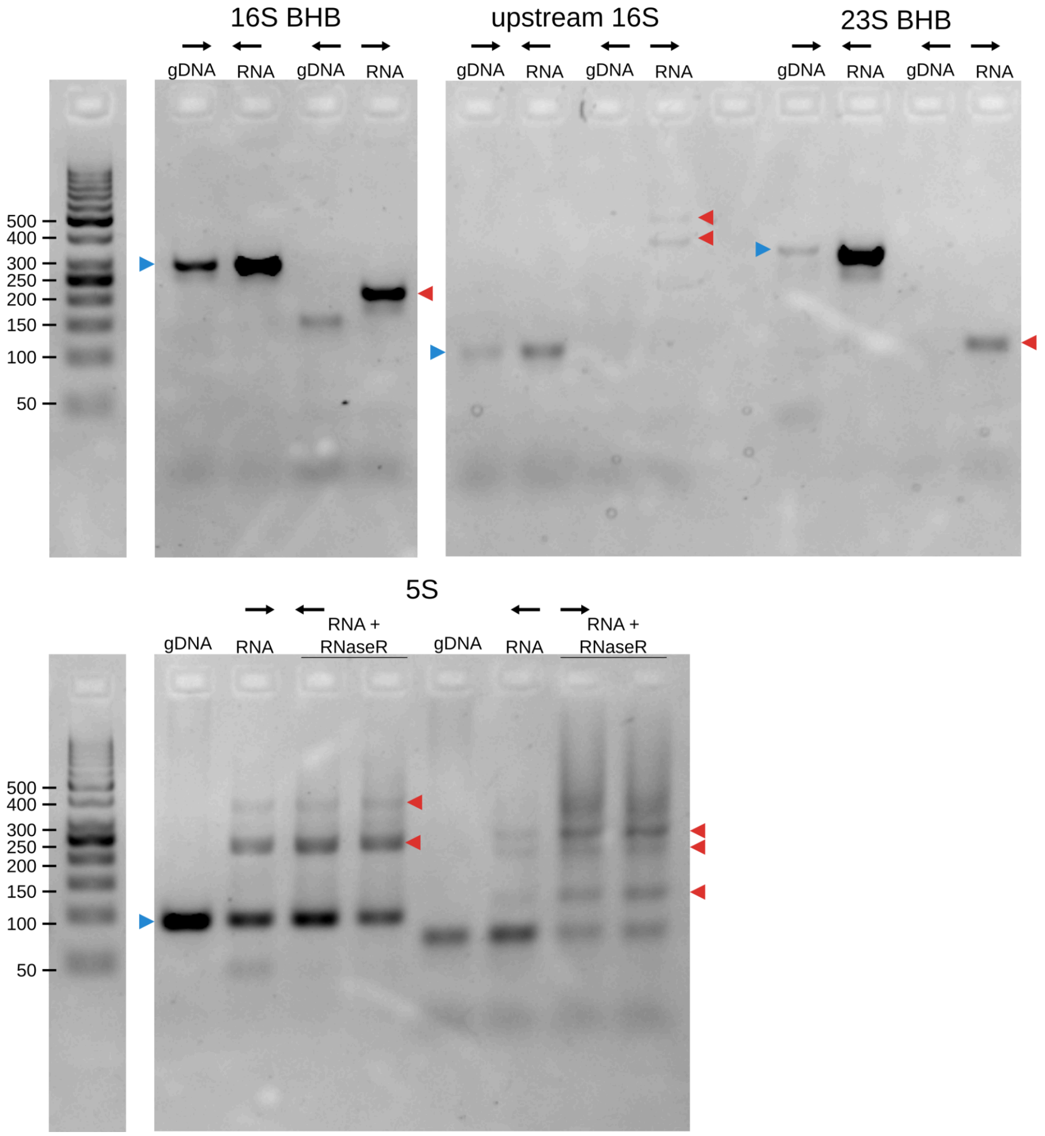

Figure S4: Uncropped gel images for rRNA circRNAs validation by RT-PCR. Convergent arrows represent reactions made with convergent primers (expected to amplify both linear and circular targets), while divergent arrows show reactions with divergent primers (expected to amplify only circular targets). Blue arrows (left) indicate the expected linear product, while red arrows (right) indicate the expected bands for circRNA junction amplification. The multiple arrows in the 16S upstream validation gel indicate expected band sizes for circRNA_0098 and circRNA_0108; multiple arrows in the 5S validation gel indicate different bands made by rolling circle amplification. Expected band sizes: 16S BHB linear - 300bp; 16BHB circular - 215bp; upstream 16S linear - 110bp; upstream 16S circular - 621bp (circRNA_0098), 374bp (circRNA_0108); 23S linear - 339bp; 23S circular - 117bp; 5S linear - 87bp; 5S circular - 84bp (circRNA_0338) or 107 (circRNA_0325). gDNA = genomic DNA; RNA = reactions made with cDNA amplified from total RNA; RNA + RNase R = reactions made with cDNA made from RNA treated with RNase R, one with total (left) and one with small RNAs (right). Thermo Scientific GeneRuler DNA Ladder 50bp was used. The image was cropped to show only lanes relevant to this figure.

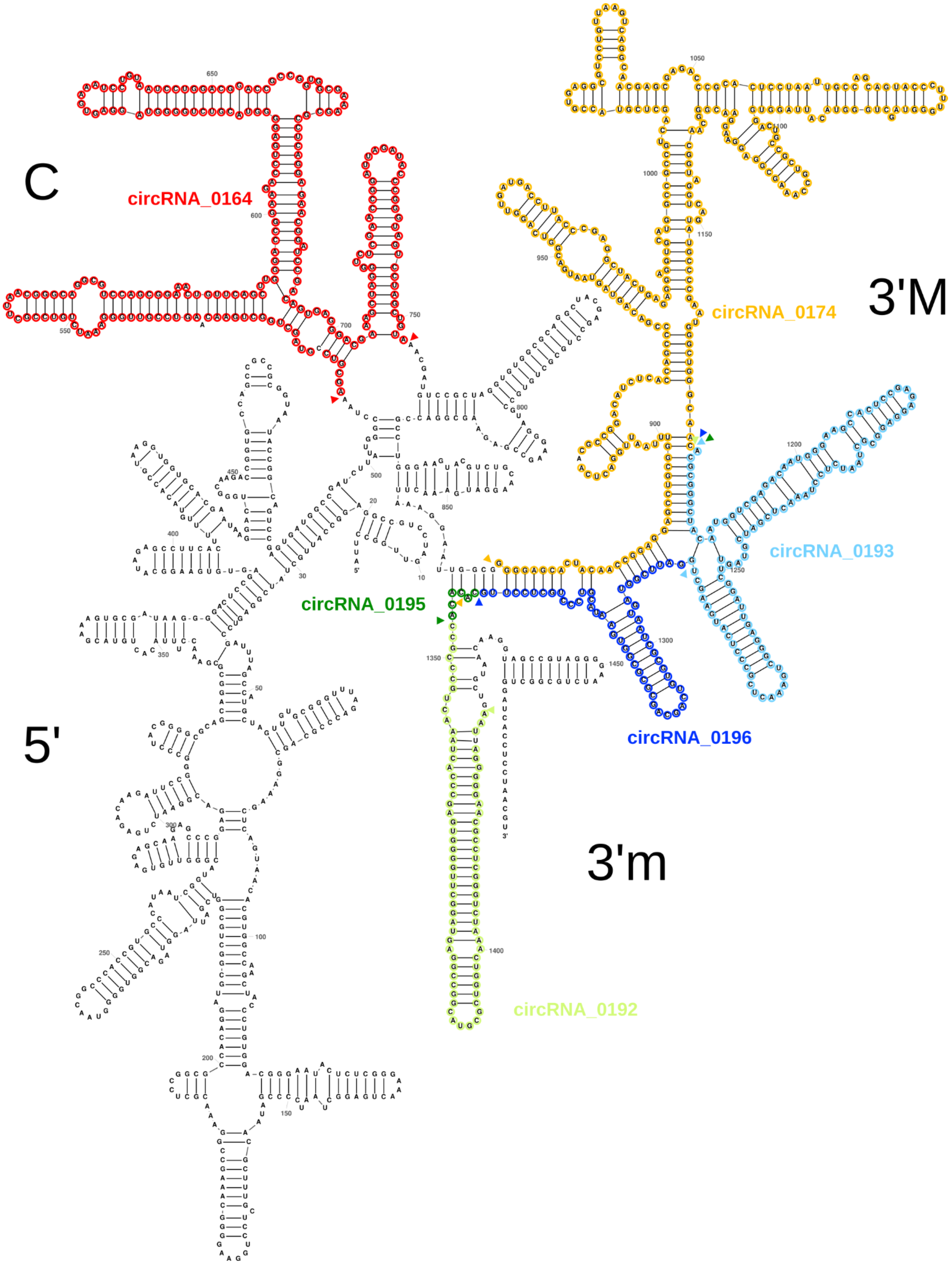

Figure S5: *Halobacterium salinarum* 16S rRNA structure. The circRNAs are highlighted in color, with the start and end coordinates of the ensemble indicated by the corresponding arrows. circRNA IDs from Table S3.

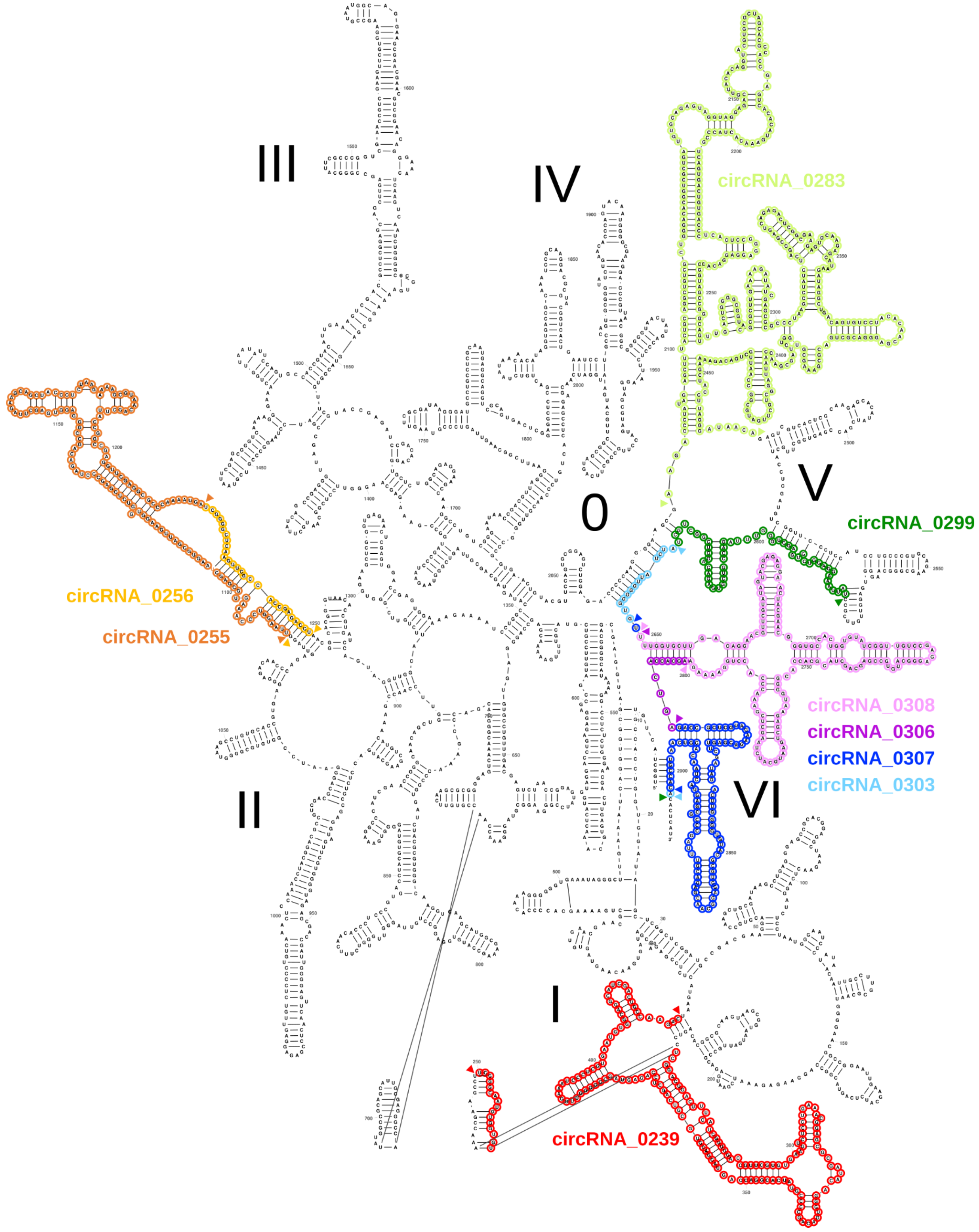

Figure S6: *Halobacterium salinarum* 23S rRNA structure. The circRNAs are highlighted in color, with the start and end coordinates of the ensemble indicated by the corresponding arrows. circRNA IDs from Table S3.

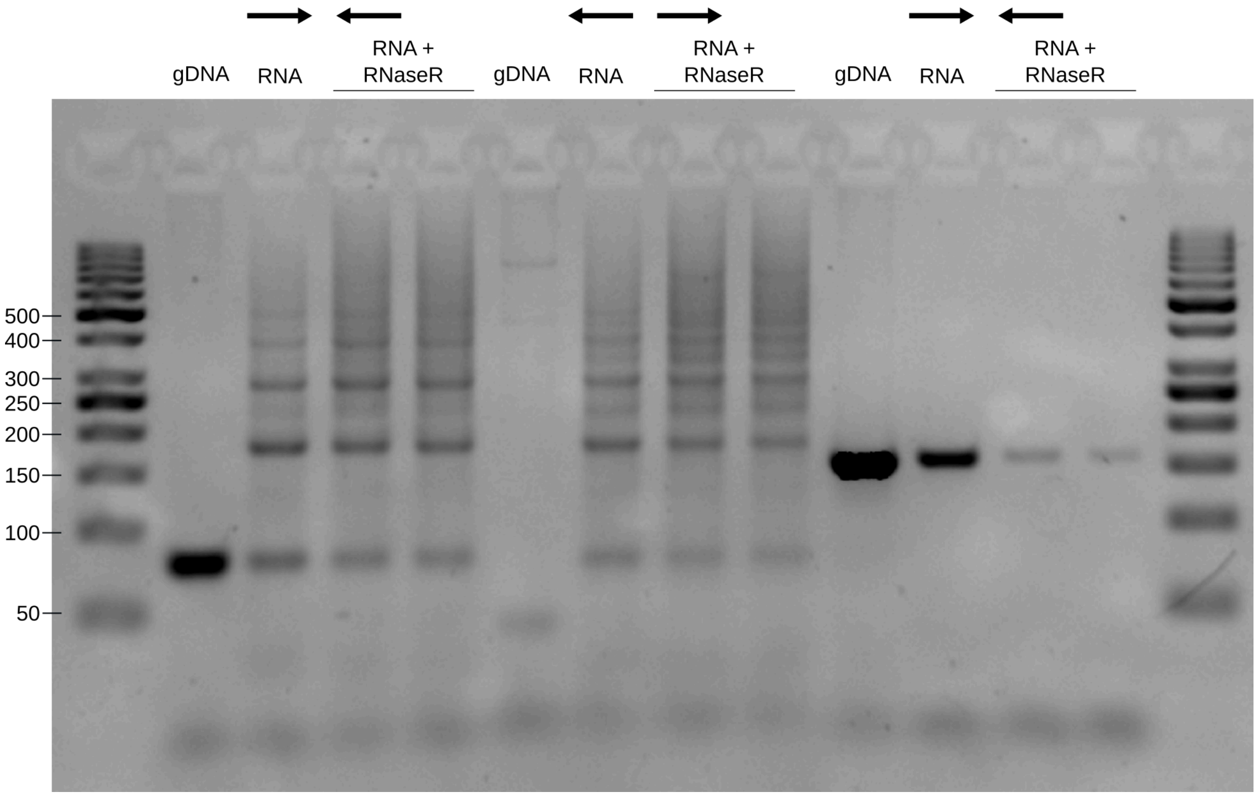

Figure S7: Uncropped gel image for circRNA_0059 validation by RT-PCR. Convergent arrows represent reactions made with convergent primers (expected to amplify both linear and circular targets), while divergent arrows show reactions with divergent primers (expected to amplify only circular targets). The convergent primers on the left lanes are inside the tRNA-Trp intron and are expected to amplify both circular and linear targets. The lanes on the right are with convergent primers that each anneal to the tRNA exons and are expected to be sensitive to RNAse R digestion and not amplify circRNAs. The multiple bands in RNA lanes are amplification products made by rolling circle amplification. Expected band sizes: linear product, convergent primers (left) - 78bp; circRNA, divergent primers (middle) - 76bp; linear product, convergent primers (right) - 157bp. gDNA = genomic DNA; RNA = reactions made with cDNA amplified from total RNA; RNA + RNase R = reactions made with cDNA made from RNA treated with RNase R, one with total (left) and one with small RNAs (right). Thermo Scientific GeneRuler DNA Ladder 50bp was used.

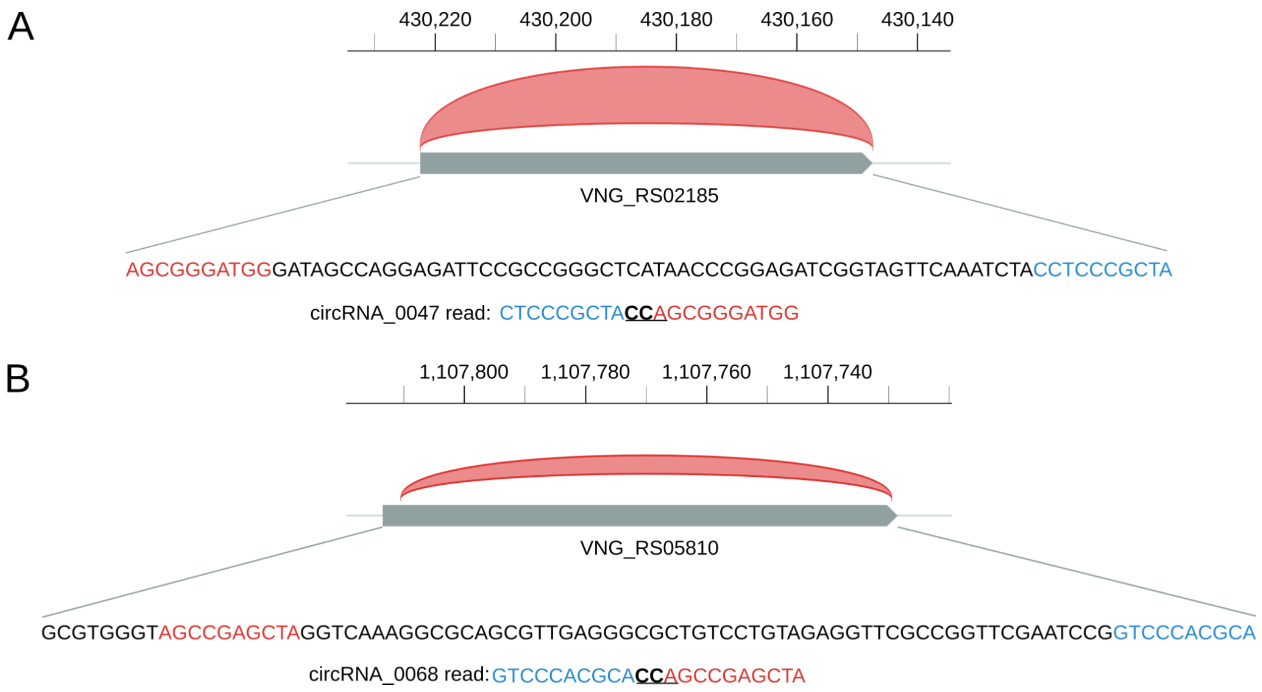

Figure S8: *Halobacterium salinarum* circRNAs that encompass whole tRNAs. (A) circRNA_0047 in tRNA-Met (VNG_RS02185). **(B)** circRNA_0069 in tRNA-Leu (VNG_RS05810). Nucleotides in bold in read sequences indicate the bases that did not align to the genome in the MonArch alignment. The sequence "CCA" is underlined.

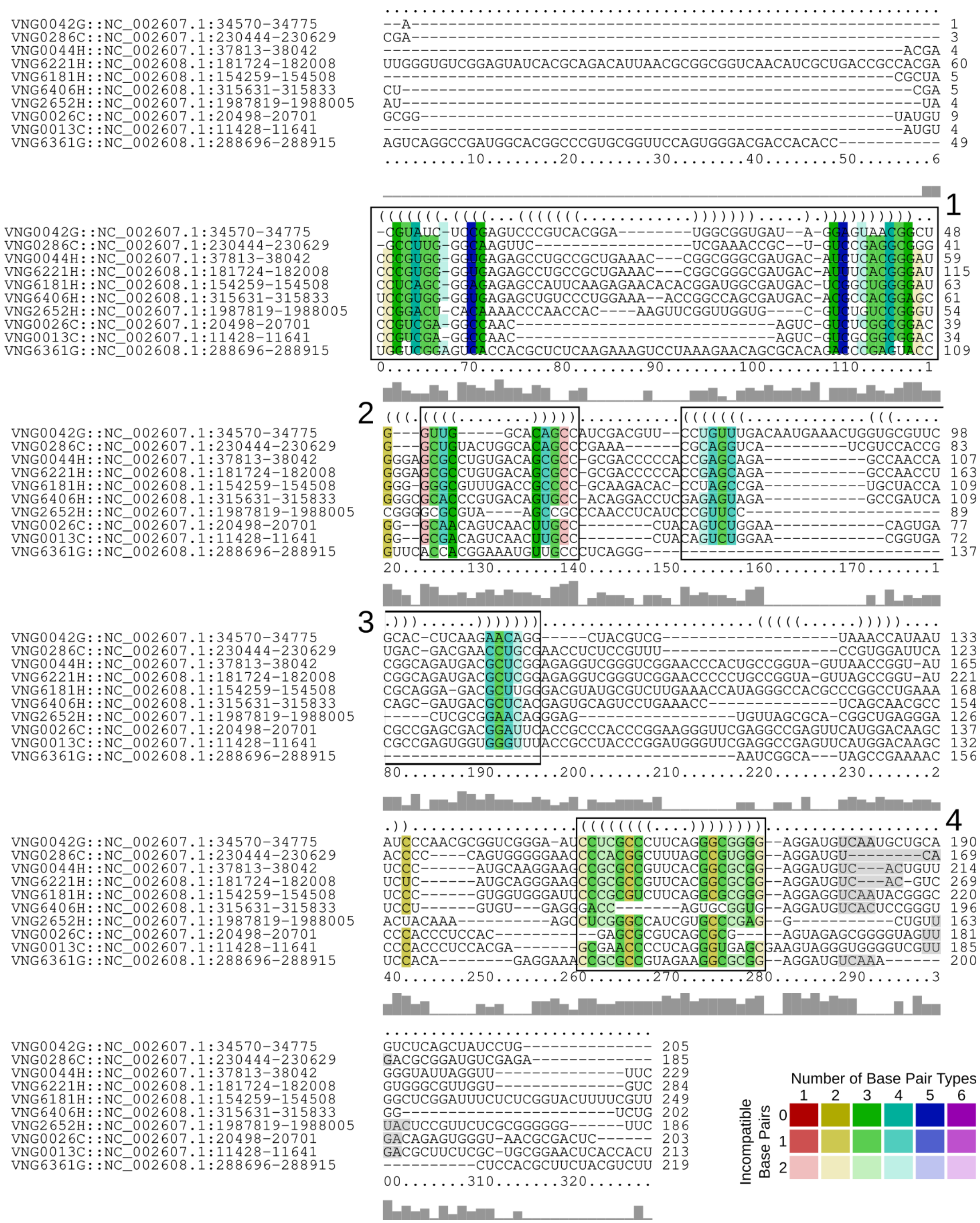

Figure S9: Multiple sequence alignment and consensus structure of the 10 *Halobacterium salinarum* ωRNAs. The structures conserved among the ωRNAs are highlighted with rectangles and numbered 1 through 4. Nucleotides with the gray background are the ones we hypothesize are the Cr/TEM (right cleavage site/transposon-encoded motif) of these RNAs (UCAA, UCAG, UCAC, UUAC, UUGA).

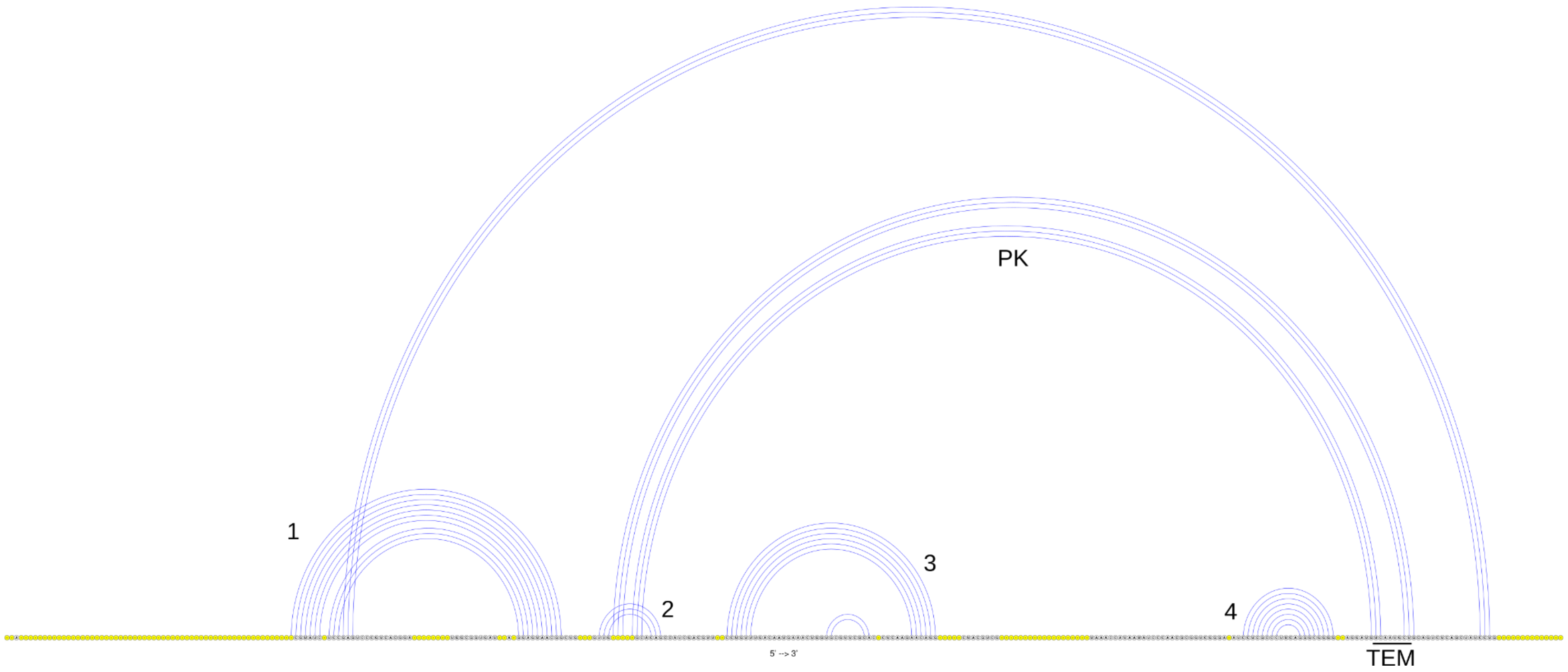

Figure S10: Pseudoknot prediction in *Halobacterium salinarum* ωRNA consensus structure. Conserved helices are numbered 1 through 4 as in Figure S9. The pseudoknot between the second helix loop and the TEM sequence (transposon-encoded motif, marked with a black bar) is indicated with "PK".

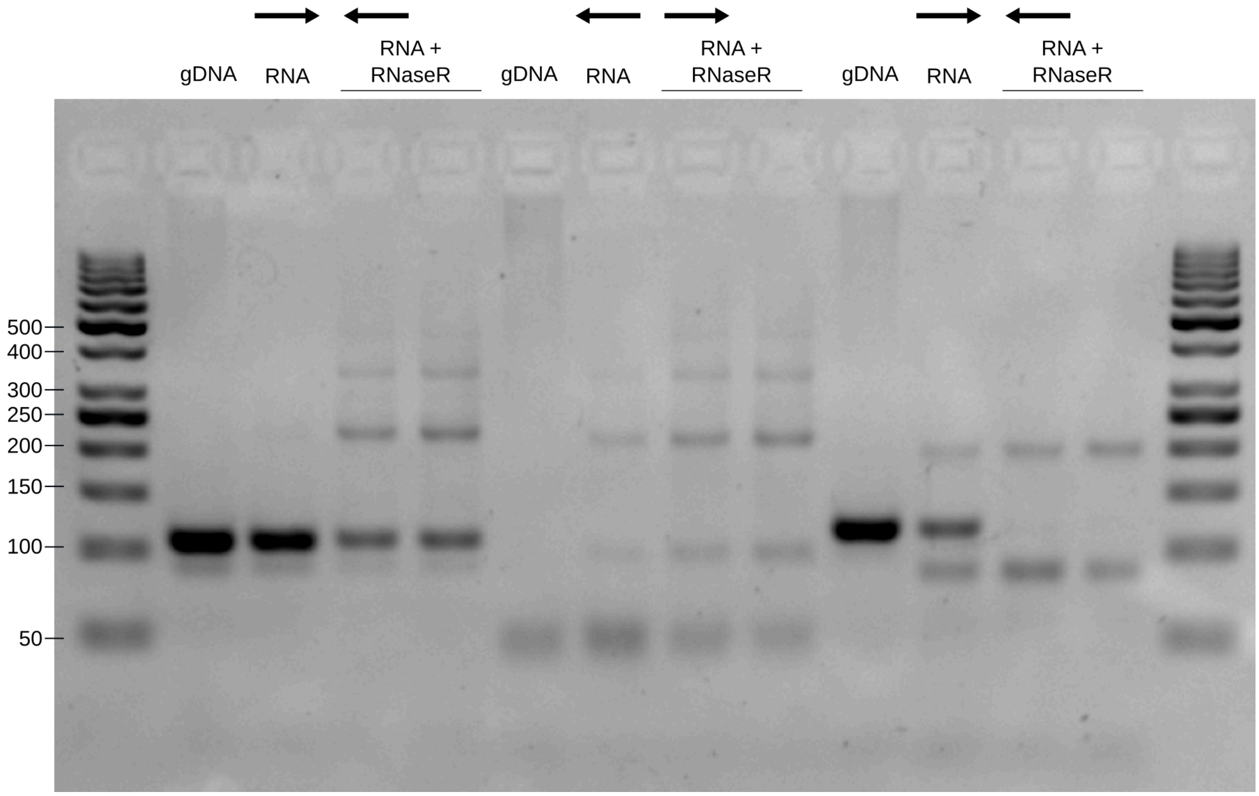

Figure S11: Uncropped gel image for circRNA_0397 validation by RT-PCR. Convergent arrows represent reactions made with convergent primers (expected to amplify both linear and circular targets), while divergent arrows show reactions with divergent primers (expected to amplify only circular targets). The convergent primers on the left lanes are inside the sot2652 ωRNA and circRNA and are expected to amplify both circular and linear targets. The lanes on the right are with convergent primers that amplify a product expected to be sensitive to RNAse R digestion and not amplify circRNAs; one primer is in the transposase gene, upstream of the sotRNA, while the other anneals to the ωRNA region. The multiple bands in RNA lanes are amplification products made by rolling circle amplification. Expected band sizes: linear product, convergent primers (left) - 109bp; circRNA, divergent primers (middle) - 100bp; linear product, convergent primers (right) - 118bp. gDNA = genomic DNA; RNA = reactions made with cDNA amplified from total RNA; RNA + RNase R = reactions made with cDNA made from RNA treated with RNase R, one with total (left) and one with small RNAs (right). Thermo Scientific GeneRuler DNA Ladder 50bp was used.

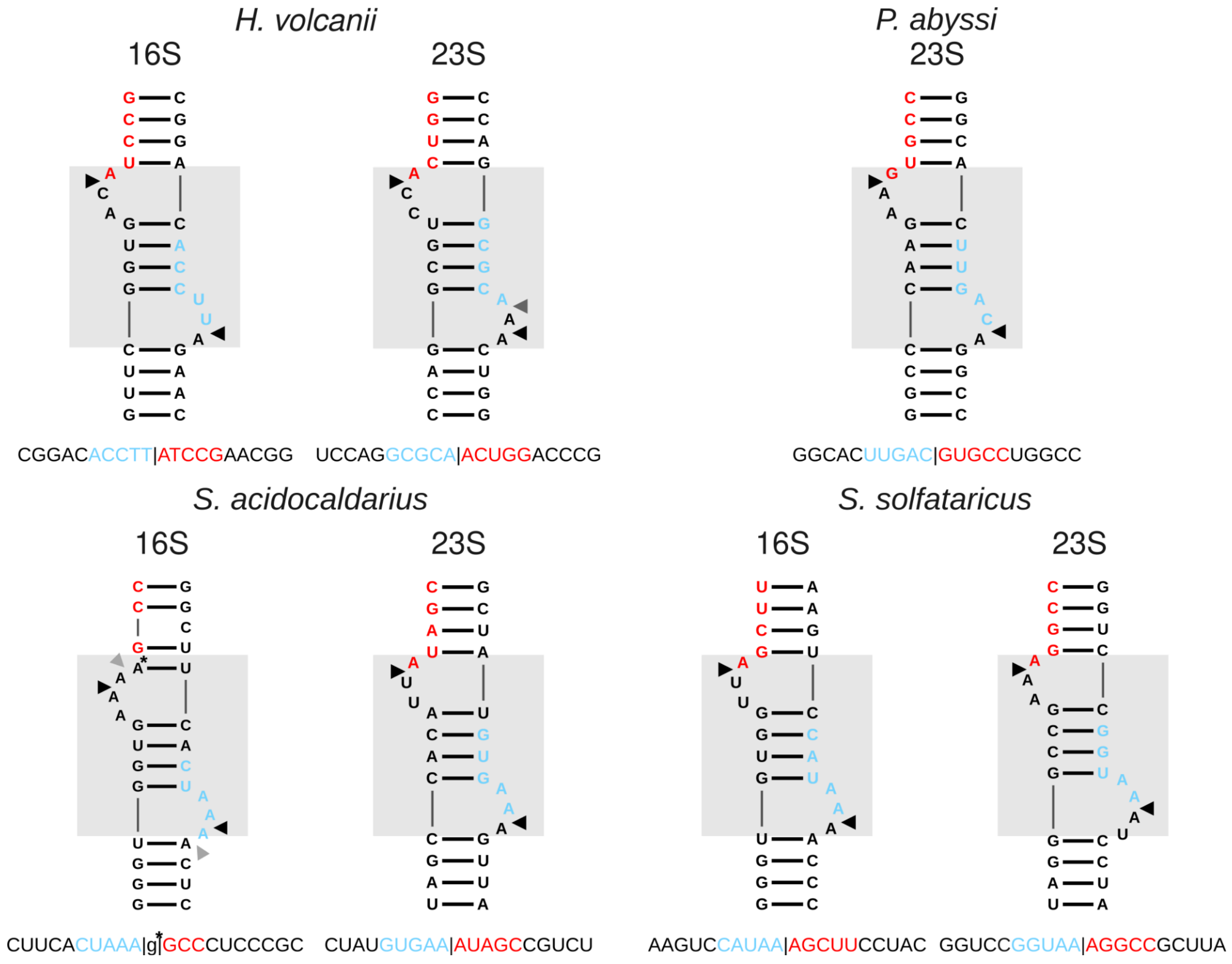

Figure S12: rRNA BHB structures and the associated circRNAs. Gray boxes indicate the BHB motif; black arrows mark the expected processing site; gray arrows indicate where we found evidence of processing based on the circularization junction reads, if different from the canonical. The sequence of the most common circularization junction sequence for these circRNAs is below each structure (circRNA_0238 and circRNA_0351 for *H. volcanii* - sequences for one of the rRNA operon, circRNA_0443 for *P. abyssi*, circRNA_3267 and circRNA_2654 for *S. acidocaldarius*, circRNA_0355 and circRNA_3125 for *S. solfataricus*). For *S. acidocaldarius*, the most common 16S BHB circularization junction had one nucleotide that did not align to the reference genome (marked with an asterisk); this could indicate a deletion or mutation in the cells sequenced.

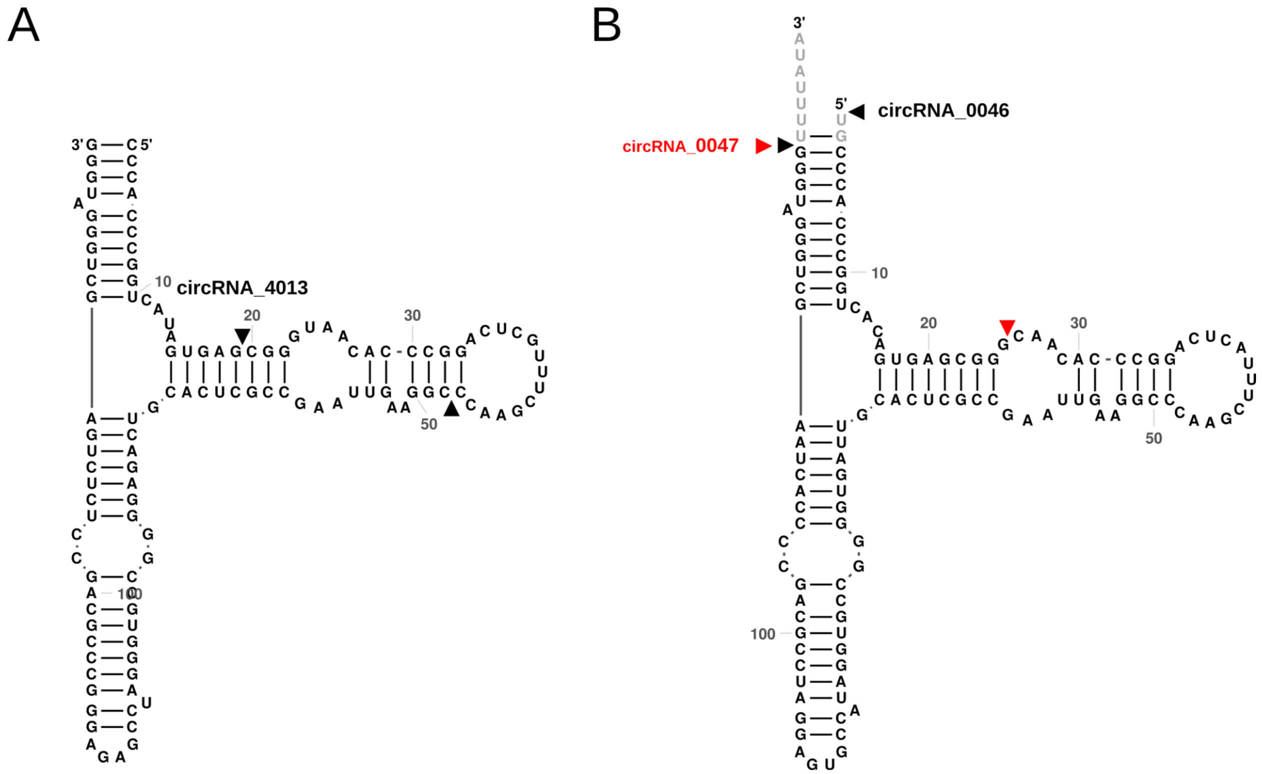

Figure S13: circRNAs in 5S rRNA of (A) *Sulfolobus acidocaldarius* and **(B)** *Saccharolobus solfataricus*. Nucleotides in black are the ones in the 5S rRNA gene; arrows mark the start and end of the most common circRNAs of the ensemble.

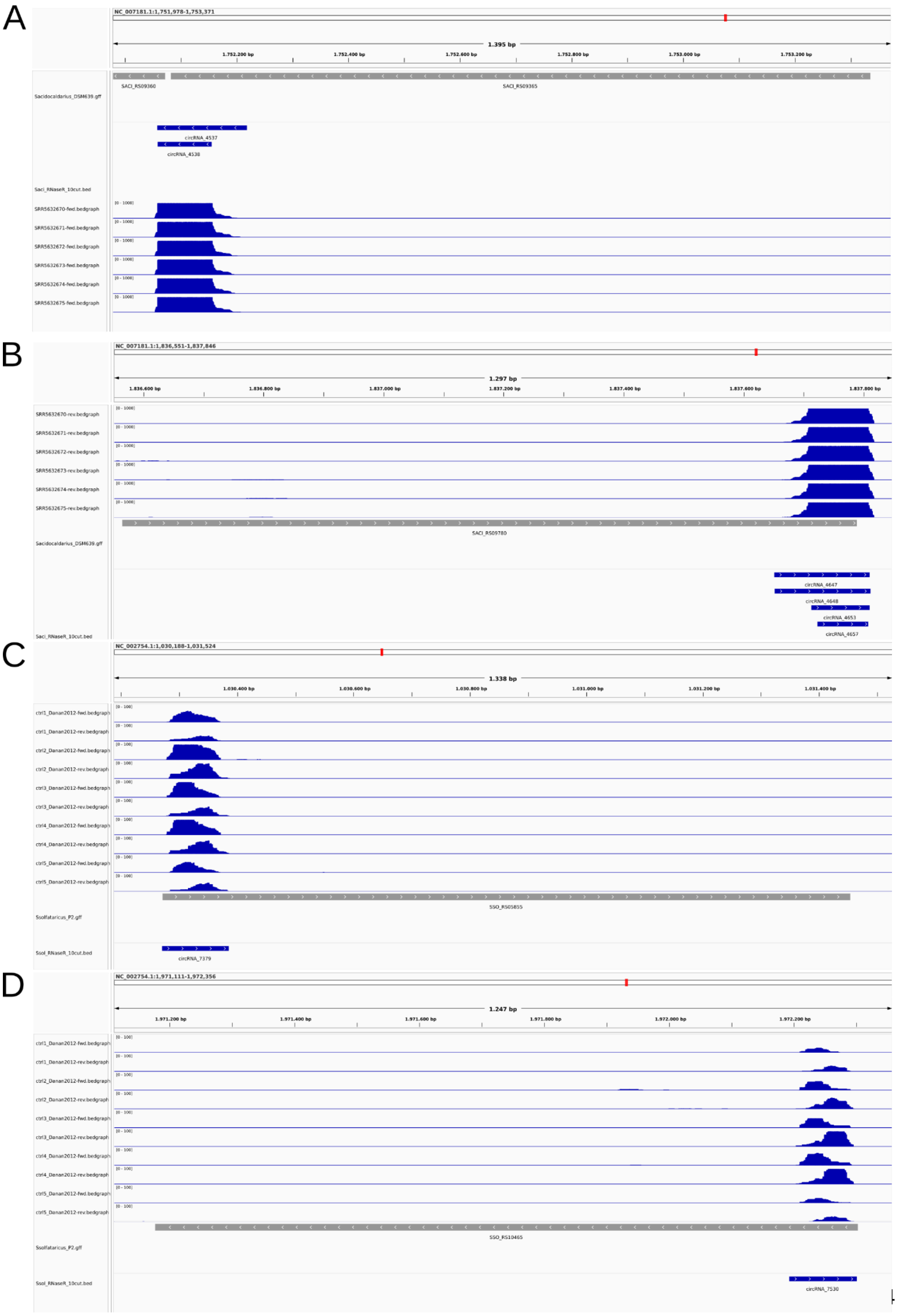
A
NC_007181.1:1,751,978-1,753,371
1.395 bp
SACI_RS09360
SACI_RS09365
Sacidocaldarius_DSM639.gff
circRNA_4537
circRNA_4538
Saci_RNaseR_10cut.bed
SRR5632670-fwd.bedgraph
SRR5632671-fwd.bedgraph
SRR5632672-fwd.bedgraph
SRR5632673-fwd.bedgraph
SRR5632674-fwd.bedgraph
SRR5632675-fwd.bedgraph
B
NC_007181.1:1,836,551-1,837,846
1.297 bp
SRR5632670-rev.bedgraph
SRR5632671-rev.bedgraph
SRR5632672-rev.bedgraph
SRR5632673-rev.bedgraph
SRR5632674-rev.bedgraph
SRR5632675-rev.bedgraph
SACI_RS09780
Sacidocaldarius_DSM639.gff
circRNA_4647
circRNA_4648
circRNA_4653
circRNA_4657
Saci_RNaseR_10cut.bed
C
NC_002754.1:1,030,188-1,031,524
1.338 bp
ctrl1_Danan2012-fwd.bedgraph
ctrl1_Danan2012-rev.bedgraph
ctrl2_Danan2012-fwd.bedgraph
ctrl2_Danan2012-rev.bedgraph
ctrl3_Danan2012-fwd.bedgraph
ctrl3_Danan2012-rev.bedgraph
ctrl4_Danan2012-fwd.bedgraph
ctrl4_Danan2012-rev.bedgraph
ctrl5_Danan2012-fwd.bedgraph
ctrl5_Danan2012-rev.bedgraph
SSO_RS05855
Ssolfataricus_P2.gff
Ssol_RNaseR_10cut.bed
circRNA_7379
D
NC_002754.1:1,971,111-1,972,356
1.247 bp
ctrl1_Danan2012-fwd.bedgraph
ctrl1_Danan2012-rev.bedgraph
ctrl2_Danan2012-fwd.bedgraph
ctrl2_Danan2012-rev.bedgraph
ctrl3_Danan2012-fwd.bedgraph
ctrl3_Danan2012-rev.bedgraph
ctrl4_Danan2012-fwd.bedgraph
ctrl4_Danan2012-rev.bedgraph
ctrl5_Danan2012-fwd.bedgraph
ctrl5_Danan2012-rev.bedgraph
SSO_RS10465
Ssolfataricus_P2.gff
Ssol_RNaseR_10cut.bed
circRNA_7530

Figure S14: circRNAs in IS200/IS605 in *Sulfolobus acidocaldarius* and *Saccharolobus solfataricus*. IGV (Integrative Genomics Viewer, Robinson et al., 2011) screenshots of IS200/IS605 transposases in *S. acidocaldarius* and *S. solfataricus* with their associated circRNAs. RNA-Seq coverage is in blue, genes are the gray boxes, and the circRNAs are the blue boxes. RNA-Seq data from the control (i.e., no RNase R treatment) samples from Orell et al., 2018 and Danan et al., 2012. **(A)** SACI_RS09365 and circRNAs circRNA_4537 and circRNA_4538. **(B)** SACI_RS09780 and circRNAs circRNA_4647, circRNA_4648, circRNA_4653, and circRNA_4657. **(C)** SSO_RS05855 and circRNA_7379. **(D)** SSO_RS10465 and circRNA_7530.
